# Pseudogene Coexpression Networks Reveal a Robust Prognostic Signature for Pediatric B-ALL Survival

**DOI:** 10.1158/2767-9764.CRC-25-0706

**Published:** 2026-04-16

**Authors:** Arturo Kenzuke Nakamura-García, Mariike L. Kuijjer, Jesús Espinal-Enríquez

**Affiliations:** 1 Computational Genomics Division, National Institute of Genomic Medicine, Mexico City, Mexico.; 2 Department of Biochemistry and Developmental Biology, University of Helsinki, Helsinki, Finland.; 3 iCAN Flagship in Digital Precision Cancer Medicine, University of Helsinki, Helsinki, Finland.; 4 Norwegian Centre for Molecular Biosciences and Medicine (NCMBM), Nordic EMBL Partnership, University of Oslo, Oslo, Norway.

## Abstract

Risk classification in B-cell acute lymphoblastic leukemia (B-ALL) remains challenging, even in the era of genomic precision medicine. Current molecular classifiers fail to fully explain the heterogeneity in patient outcomes, suggesting that key regulatory layers remain hidden. In this study, we uncover a previously unexplored dimension of B-ALL biology by analyzing coexpression patterns between pseudogenes using single-sample coexpression networks (*n* = 1,416). Principal component analysis showed that these interactions explain a major component of variability among patients and contribute to patient stratification into clusters with distinct overall survival. After identifying interactions associated with these clusters, we used a LASSO-based feature selection pipeline to derive a three-interaction signature that predicted patient survival, with *RPL7P10*–*RPS3AP36* emerging as the most robust biomarker. Our study shows that coexpression between pseudogenes represents a previously unrecognized layer of molecular heterogeneity in B-ALL, harboring promising molecular markers for future studies.

**Significance:**

This study reveals pseudogene coexpression as a previously unrecognized driver of transcriptional heterogeneity in B-ALL. We identify robust survival biomarkers derived from these interactions and introduce a single-sample network framework that enables precise patient stratification and biomarker validation in independent cohorts.

## Introduction

B-cell acute lymphoblastic leukemia (B-ALL) is a type of hematologic malignancy characterized by a clonal expansion of malignant B-cell progenitors. This type of leukemia is the most frequent among pediatric patients, and although it has high cure rates, relapse is still very common (1–3). Next-generation sequencing has helped to uncover new B-ALL subtypes, which are mostly defined by gene fusions and chromosomal rearrangements (4–9). However, these works have mainly focused on deregulation among protein-coding genes, leaving the potentially oncogenic roles of noncoding DNA sequences largely unexplored. Recent studies have started to explore the potential regulatory roles of pseudogenes, suggesting that they may contribute to the deregulation of the gene expression landscape observed in complex diseases such as B-ALL (10).

A pseudogene is a copy from a protein-coding gene that, due to detrimental alterations in its sequence, has lost its ability to code the original functional protein (11). Historically, these types of DNA sequences have been considered “junk” DNA, and hence, research in their possible roles in gene regulation has been largely hampered. However, it has been demonstrated that pseudogenes can be transcribed and even translated, although their functions diverge from their parental coding gene (12). Multiple works have demonstrated that pseudogenes are actively involved in gene regulatory circuits through diverse mechanisms. For example, they can alter the translation of their parental gene through endogenous competition for regulatory elements or even alter DNA structure to promote or repress the transcription of a sequence (reviewed in ref. 11). Moreover, pseudogenes have been found to function as important modulators of gene expression in cancer (13). Given the regulatory role of pseudogenes in gene expression, understanding their interactions within the coexpression landscape can provide valuable insights into the underlying biological processes in which they are involved. Furthermore, analyzing them in the context of complex diseases, like B-ALL, can reveal novel information about the regulatory rewiring that drives disease pathology.

Gene coexpression networks (GCN) are a common tool in the field of systems biology to approach the inherent complexity of biological systems (14). These networks are theoretical graphs composed by genes connected between them when their expression profiles are correlated. A coexpression interaction between a pair of genes suggests a coordination which is possibly part of the same biological process (15). Previous works have described GCNs from multiple cancer types (16–26).

We have reported an increased coexpression between pseudogenes in hematologic cancers compared with normal samples (27). This suggests that a coordination between these sequences could be involved in the biology of these malignancies, which warrants further exploration. However, these recent results were obtained through the analysis of aggregate GCNs, constructed by integrating data from multiple samples. Consequently, these pseudogene coexpression networks provide only a summary of the biological data across populations. As a result, the aggregate networks fail to capture the intrinsic biological heterogeneity within the population.

Biological samples are highly heterogeneous; understanding how their inherent variability affects biological systems is crucial for treatment design or when evaluating patient clinical features (such as survival rates). In general, a one-size-fits-all approach is inadequate for addressing complex diseases (28, 29), necessitating a more personalized approach.

Because of the latter, in the context of systems biology, new approaches have been developed to construct GCNs at the single-sample resolution. These approaches enable the creation of “single-sample networks” (SSN), which describe the coordination of genes within individual samples from a population. Various methods have been proposed for this purpose (reviewed in ref. 30), each differing in how the individual sample networks are calculated. One such method is LIONESS (31), which uses a leave-one-out strategy to estimate each sample’s contribution to the aggregate network. This method has been successfully applied to understand gene regulatory mechanisms in various cancers, leading to the identification of potential new regulatory subtypes in leiomyosarcoma (32), sex-biased regulatory patterns affecting prognosis in colon cancer (33), regulatory signatures associated with poor prognosis in glioblastoma (34), and subtype-specific coexpression hotspots in breast cancer (35).

Motivated by our previous observation of increased pseudogene coexpression in leukemias (27), we hypothesized that modeling these interactions at the single-sample level could reveal heterogeneity associated with clinical outcomes in B-ALL. To test this, we applied the LIONESS algorithm to construct single-sample coexpression networks from RNA-seq data of 132 patients from the TARGET-ALL-P2 cohort, focusing exclusively on pseudogene–pseudogene interactions. Based on edge weights from these individualized networks, we clustered patients, finding that pseudogene coordination patterns alone were sufficient to stratify patients into groups with significantly different overall survival (OS). Importantly, this signal was not recoverable when pseudogene interactions were excluded from the analysis.

We prioritized the most prognostically informative edges through a resampling-based LASSO strategy applied to the combined dataset of two cohorts (TARGET-P2-ALL and MP2PRT-ALL), which consistently selected three stable interactions. A multivariate Cox model built on these interactions, trained exclusively in TARGET and evaluated in MP2PRT, retained predictive performance and achieved significant OS stratification across cohorts. It is important to mention that MP2PRT-ALL cohort is enriched for patients with standard risk and high risk with genetic features associated with favorable outcome, with only ∼6% mortality within 5 years.

To test whether the observed generalizability of the model was driven by a true biological signal or was merely an artifact of the feature selection pipeline, we generated 1,000 null models with randomly selected features, applying the same LASSO-based filtering and evaluation procedures. Only one of these null models outperformed the real model, highlighting the biological relevance of the selected pseudogene interactions.

Among the selected features, the interaction between *RPL7P10* and *RPS3AP36* stood out as the most consistent and predictive. We evaluated its prognostic value independently and showed that it was sufficient to significantly stratify patients by survival risk in both datasets.

Overall, these results highlight pseudogene coexpression as a potential source of novel survival biomarkers in cancer and underscore the value of sample-level network approaches for advancing personalized medicine in B-ALL.

## Materials and Methods

The code for reproducing the results of this work can be found at: https://github.com/AKNG97/PSs-SSNs-in-B-ALL.git. Every step of the pipeline was executed in R.

### Data acquisition

The RNA sequencing (RNA-seq) data used in this work were retrieved from Genomic Data Commons (GDC, https://portal.gdc.cancer.gov). Expression data from B-ALL samples were obtained from the MP2PRT-ALL and TARGET-ALL-P2 projects. The study was based exclusively on publicly available, de-identified data. All original sample collection procedures were conducted in accordance with the relevant ethical guidelines and approved by the corresponding institutional review boards. No additional ethical approval or informed consent was required for this secondary data analysis. Only cancer samples categorized as “primary blood-derived cancer–bone marrow” with complete survival follow-up information were included. Normal bone marrow samples were obtained from the TARGET-AML project. The types of counts used included TPM and unstranded counts.

A complementary gene annotation file was obtained from Biomart (36).

### Data preprocessing

Unstranded counts from cancer and normal samples were processed together. Only genes present in both the expression matrix and the external gene annotation were considered. All genes with duplicated HGNC symbols were removed. When multiple replicates of a sample were found, we summed the expression of genes between replicates with the function “collapseReplicates” from the “DESeq2” (37) package. Next, we filtered out lowly expressed genes in the following order:

1. Genes on the first quartile of mean expression.
2. Genes with 0 counts in at least 50% + 1 samples.
3. Genes with average expression less than 10.

This first filtering method substantially reduces the likelihood that spurious correlations arise from sparse or near-background expression levels.

### Network inference

For the coexpression analysis, raw counts were normalized with the following pipeline:

1. Within-sample normalization by gene length and GC content with the EDASeq package (38).
2. Between-sample normalization by trimmed mean of M values (TMM) with the NOISeq package (39).
3. Batch effect removal by ARSyN from the NOISeq package (39).

Gene length and GC content were retrieved from Biomart. Normalized counts from cancer samples were used to calculate the aggregate GCN using the Spearman rank correlation coefficient. Correlations with a *P* value < 10^−8^ were considered statistically significant. The LIONESS equation (31) was applied on the aggregate GCN to obtain the single-sample edge weights.

### Sequence similarity analysis in pseudogene families

The analysis between pseudogenes and parental genes used the TPM counts for the TARGET B-ALL samples. Sequences were obtained from Biomart (36) using the function “getSequence” by Ensembl IDs. The type of sequence retrieved was “gene exon intron.” Sequence similarity scores were calculated using the Smith–Waterman algorithm with the function “smith waterman” from the “text.alignment” package (40).

For the analysis of sequence similarity between pairs of pseudogenes, we analyzed communities from the pseudogene-based networks (*PG*_*net*_) identified through the Louvain algorithm [from the “igraph” (41) package] to generate the communities.

### Clustering analysis

Hierarchical clustering analysis was performed using the M3C package. The function uses a Monte Carlo simulation to generate a null distribution of stability scores while preserving the structure of the input data. From this, a relative clustering stability index (RCSI) is calculated to evaluate the plausibility of different numbers of partitions (K) in the data compared with the null hypothesis that the data contain no clusters (K = 1; ref. 42). The function returns an optimal value of K using the RCSI and its associated *P* value.

M3C was run with a set of top variable features normalized to *Z*-scores across samples. For the analysis of the *PG*_*net*,_ we used the top 25% most variable edges. Then, for the analyses of the complete network and the network without PS–PS edges, we used the same amount of top variable features as in the analysis of the *PG*_*net*_. PCA analysis of input data is computed automatically in each run of M3C.

### Differential coexpression analysis

Differential coexpression analysis was performed by the median differences between groups. The significance of these differences was assessed using the Wilcoxon test. *P* values were adjusted for multiple testing using the false discovery rate (FDR) method. Edges with an absolute median difference greater than 0.5 and an FDR <0.05 were considered differentially coexpressed.

### Projection of the MP2PRT-ALL single-sample coexpression values into the TARGET-ALL-P2 LIONESS model

LIONESS models estimate the contribution of individual samples to the global coexpression pattern by leveraging the variability present in a reference population. However, LIONESS-derived networks are not directly comparable when computed independently across different datasets.

Although one potential strategy for comparison would be to combine all samples into a single LIONESS model, this approach would be suboptimal in our context due to the distinct clinical composition of the TARGET and MP2PRT cohorts. Integrating both datasets into a unified model would introduce a substantial bias toward the coexpression patterns of MP2PRT, given its larger sample size. Moreover, such integration would distort the interpretation of LIONESS edge weights derived from the original, independent models, complicating their biological interpretation and undermining the comparability of the results across cohorts. For instance, it would not be possible to determine whether biomarkers identified in one cohort are also equally relevant in the other, as the combined model would alter the underlying edge weight distributions and their interpretation.

To overcome this limitation, we simulated an *in silico* clinical setting by projecting each MP2PRT sample into the coexpression space defined by the TARGET dataset. Specifically, for each MP2PRT sample, we normalized its raw expression counts using the raw counts from TARGET and then recalculated the LIONESS model incorporating that sample alongside the TARGET reference. This process was repeated independently for each MP2PRT sample to ensure the overall structure of the TARGET-based model remained stable. The resulting edge weights represent how each MP2PRT sample aligns with the TARGET coexpression landscape and are referred to as “projected” edge weights. By doing so, all samples are evaluated within a common reference framework, enabling a consistent comparison across cohorts while preserving the original interpretation of LIONESS edge weights.

### Feature selection and multivariate model construction

To build a minimal and robust predictive model for survival, we performed a Lasso regression using the 42 edges that were differentially coexpressed between cluster 1 (associated with good survival) and clusters 2 and 3 (associated with poor survival) in the TARGET analysis.

To increase input variability, we included the projected MP2PRT edge weights alongside the original TARGET edge weights in a combined dataset. We then generated 100 random partitions, each including 50% of the total samples, and performed LASSO feature selection independently in each partition. This strategy served two complementary purposes.

First, randomly combining both datasets increased the overall variability in coexpression patterns, reducing the risk of overfitting to cohort-specific noise and enabling the identification of features informative across a broader range of biological and clinical heterogeneity.

Second, by incorporating MP2PRT samples into the selection process, we aimed to identify coexpression edges that were not only statistically robust within the TARGET cohort but also stable under variation introduced by an independent population. In this sense, the combined selection process served as a filter to prioritize edges with greater potential to generalize beyond the original training cohort.

Finally, edges were ranked according to their selection frequency across the 100 LASSO runs. We then examined the change in frequency between consecutively ranked features to identify the first major inflection point in selection stability. This inflection occurred at the fourth-ranked position, in which two edges tied with a selection frequency of 76. The drop from the first to the second and from the second to the third ranked edges was 3 in both cases, reflecting a stable decline. In contrast, the drop from the third to the fourth position was 5, representing a 1.67-fold increase in the magnitude of the frequency drop. Features ranked above this inflection point were retained for model construction, thereby minimizing the inclusion of unstable edges.

Although MP2PRT samples contributed to the feature selection process, the final multivariate Cox proportional hazards model was trained exclusively on TARGET samples, ensuring that model fitting remained independent from the evaluation cohort.

### Multivariate model performance evaluation

To evaluate the predictive power of the final model, we first used it to calculate risk scores for each sample in both the TARGET and MP2PRT cohorts. To define high- and low-risk groups, we applied a fixed threshold corresponding to the median risk score observed in the TARGET cohort. This approach ensured consistency in group assignment across datasets and avoided information leakage from the validation cohort. Additionally, using a fixed decision boundary allowed us to directly assess whether the survival-associated patterns identified in TARGET generalized to MP2PRT. We then computed time-dependent ROC–AUC values at 5 years to evaluate the model’s predictive accuracy in both cohorts.

Because the MP2PRT cohort was included in the feature selection process, we sought to determine whether the model’s performance in MP2PRT could be attributed to selection bias rather than to a truly generalizable biological signal. To address this, we constructed 1,000 null models by randomly selecting 42 edges from the full coexpression network defined in the TARGET dataset. For each null model, the projected edge weights for MP2PRT were computed, and the combined TARGET + MP2PRT dataset was subjected to the same LASSO-based feature selection procedure used in the real model, with 100 random partitions each including 50% of the samples.

Within each null model, edges were ranked by their selection frequency, and the drop in frequency between consecutively ranked edges was used to identify the first major inflection point in selection stability. Specifically, we computed the ratio between successive drops and retained all edges ranked above the point in which the drop magnitude increased by at least 1.67-fold compared with the previous drop. This threshold was empirically derived from the behavior observed in the real model, in which the top three features showed regular frequency drops of 3, followed by a sharper drop of 5 at the fourth position (5/3 = 1.67), marking the first disruption in selection stability. The same 1.67 ratio was applied across all null models to ensure consistency and comparability in feature selection.

A multivariate Cox proportional hazards model was then trained using only TARGET samples and the selected edges from each null model. Risk scores were calculated in both cohorts using the trained model, and samples were classified into high- and low-risk groups using the same median threshold defined in TARGET. Each null model was evaluated using the Kaplan–Meier analysis, in which a model was considered significant if the analysis showed a *P* < 0.05 and the risk groups preserved the expected survival direction (i.e., higher survival in the low-risk group) across cohorts. Finally, to compare the overall performance of the null models against the pseudogene-based model, we analyzed the models’ 5-year ROC–AUC values. Overall, this analysis showed that only 48 random models managed to generalize from TARGET into MP2PRT, and only one of them outperformed the real model by its 5-year ROC–AUC values.

This analysis enabled us to compare the real model’s performance with the distribution of null models under the same selection and evaluation criteria and determine whether the observed generalization in MP2PRT could be an artifact of the feature selection process.

### *RPL7P10*–*RPS3AP36* predictive analysis

To assess the individual contribution of the edges included in the multivariate model, we independently evaluated their predictive performance. Specifically, we computed time-dependent ROC–AUC values from years 1 to 5 for each edge. This analysis revealed that the interaction *RPL7P10*–*RPS3AP36* was the most stable and consistent predictor of OS across both cohorts.

To further evaluate the predictive value of this edge, we stratified the TARGET cohort into high- and low-risk groups using its edge weights. As this analysis was based on a single feature, we hypothesized that a threshold informed by underlying biological structure, rather than the median weight from the full distribution, would better distinguish the risk groups. For this purpose, we used the median edge weight observed within cluster 1 (low-risk group) from the TARGET stratification. Based on our previous observations, we hypothesized that this value (−0.741) reflects a biologically relevant boundary that separates different coordination states associated with distinct clinical outcomes. This threshold was applied to both TARGET and MP2PRT (using projected edge weights) to define risk groups, which were then evaluated by Kaplan–Meier analysis for OS.

### Transcription factor binding site enrichment analysis

In order to identify potential transcription factors (TF) involved in the regulation of relevant pseudogenes, we performed an enrichment analysis using UniBind (43, 44). UniBind is a map of direct TF–DNA interactions derived from chromatin immunoprecipitation sequencing peak datasets and allows the comparison of genomic regions to identify potential regulatory sequences. The database was accessed through https://unibind.uio.no/, and the enrichment analysis was performed using BED files containing the coordinates of the 1,000 bp upstream and 250 bp downstream regions from the transcription start site of each gene.

Specifically, we enriched the regions of *RPL7P10* and *RPS3AP36*, using as background all pseudogenes present in the *PG*_*net*_. The BED files were constructed by retrieving the start and end coordinates of each gene from BioMart (36). Finally, we compared the expression distributions of TFs with enriched binding sites (TFBS). For this purpose, raw expression counts from each cohort were normalized separately using the TMM method implemented in the NOISeq package (39). Wilcoxon tests were performed between groups for each cohort and TF, and *P* values were adjusted using the FDR method.

## Results

### Pseudogene coexpression landscape reveals interchromosomal and family-driven coordination

To characterize the transcriptional coordination among pseudogenes in B-ALL, we constructed an aggregate coexpression network using RNA-seq data from 132 patients of the TARGET-ALL-P2 cohort (see “Materials and Methods”). The expression matrix used for this analysis contained 22,281 genes, from which the major types of RNA were protein-coding genes (15,692), pseudogenes (2,877), and long non-coding genes (2,452). The median expression of pseudogenes was approximately half that of protein-coding genes in B-ALL (pseudogene to protein-coding ratio = 0.50), whereas in normal bone marrow, this ratio was lower (0.39), indicating an overall increase of pseudogene transcription in leukemia (Supplementary Table S1).

**Figure 1.**
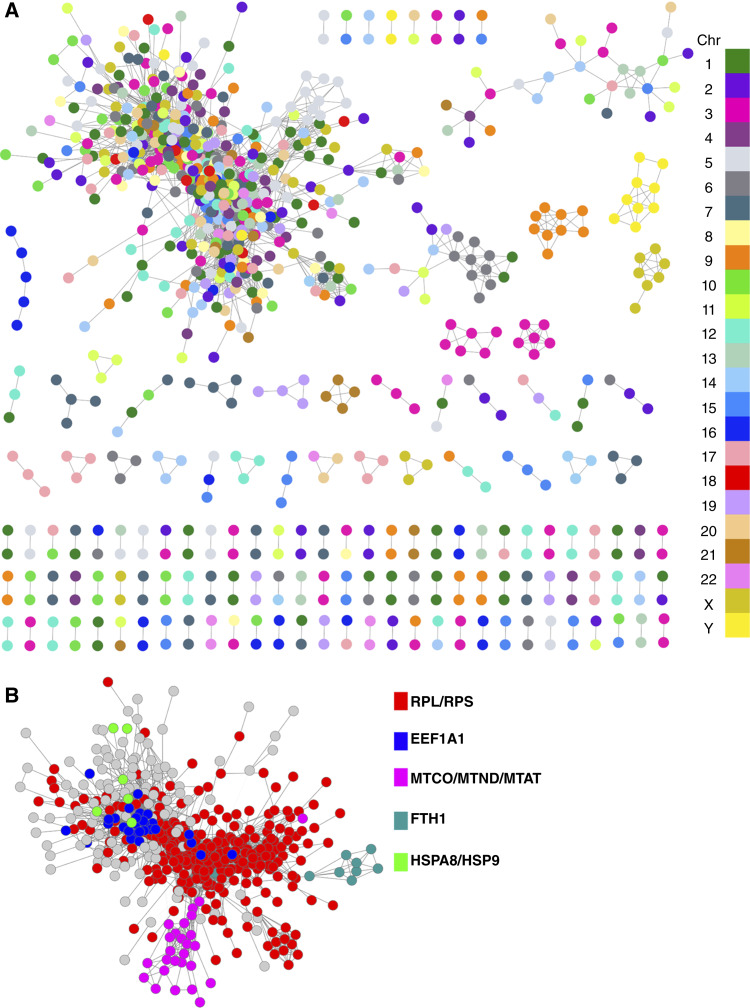
**A,** Aggregate gene coexpression network between pseudogenes (*PG*_*net*_) in B-ALL samples. The color of nodes corresponds to the chromosome in which those pseudogenes are located. To note, the largest component contains pseudogenes from all chromosomes, whereas the smaller ones are mostly formed of pseudogenes from the same chromosome. **B,** Largest component of (**A**), with pseudogenes colored according to their families.

For network construction, edges with a correlation *P* value below 10^−8^ were considered significant. The resulting graph included 45,114 edges and 11,379 genes, of which 6,032 edges connected 865 pseudogenes. This pseudogene coexpression network, referred to hereafter as the *PG*_*net*_, forms the foundation for all subsequent analyses (Fig. 1). The correlation and expression values of genes in the top 10 coexpression interactions are shown in Supplementary Table S2.

In Fig. 1A, nodes correspond to pseudogenes and are colored according to their chromosome of origin. Whereas smaller subgraphs are composed predominantly of intrachromosomal edges, the largest component integrates pseudogenes from all chromosomes, suggesting broad interchromosomal coordination. However, when pseudogenes are colored by family (Fig. 1B), a clear clustering by parental origin emerges, with 94.6% of interactions in the largest component occurring between pseudogenes from different chromosomes but belonging to the same or closely related gene families.

Given the sequence similarity between pseudogenes and their parental genes (45), we evaluated whether the observed network structure could be explained by misalignment artifacts in RNA-seq. To test this, we compared expression correlations and sequence similarity across pseudogene families (Supplementary Fig. S1A; Supplementary Table S3). Fourteen families showed moderate associations (Supplementary Fig. S1B–S1D); however, highly similar pseudogenes were frequently expressed at low levels (TPM < 1; Supplementary Fig. S1E). When these low-expression members were excluded, correlations weakened substantially and family sizes decreased (Supplementary Fig. S1F), indicating that alignment artifacts are unlikely to account for the global coordination observed in the *PG*_*net*_. Moreover, coexpression edge weights were essentially independent of sequence similarity between pseudogene pairs (Supplementary Fig. S2A–S2H), further supporting the biological relevance of the network.

### Individualized *PG*_*nets*_ stratify patients with B-ALL into survival-relevant clusters

To explore interpatient heterogeneity in pseudogene coordination, we applied the LIONESS algorithm (31) to construct SSNs from the *PG*_*net*_ for each of the 132 patients in the TARGET cohort. This approach estimates the contribution of each individual sample to the global coexpression network by calculating the difference in correlation values when the sample is excluded. The resulting SSNs allow the identification of patient-specific regulatory patterns, enabling fine-grained comparisons between individuals.

To identify subgroups of patients with similar pseudogene coexpression profiles, we used the M3C clustering algorithm, which estimates an RCSI and evaluates significance against a reference null distribution (42). Because M3C recommends to use a set of variable features that are approximately normally distributed, we first filtered the top 25% most variable edges from the *PG*_*net*_ and then scaled these edges to *Z*-scores across samples (by subtracting the mean edge weight from the sample weight and dividing it by the edge’s standard deviation).

**Figure 2.**
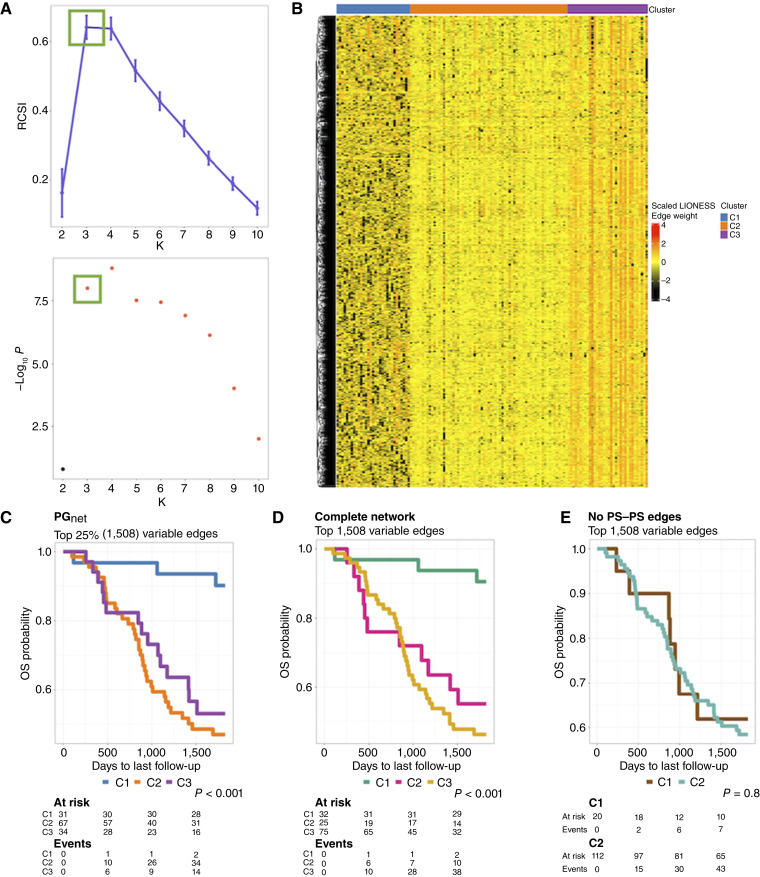
Clustering analysis of SSNs of B-ALL samples from TARGET-ALL-P2. **A,** The M3C algorithm shows K = 3 as the optimal partition for the data. The top figure shows the RCSI scores calculated for up to 10 partitions of the dataset. The bottom figure shows the *P* values associated with each K of the top figure. **B,** Heatmap showing the SSN values scaled across samples clustered according to the M3C result. **C,** Kaplan–Meier analysis in the *PG*_*net*_. **D,** KM plot in the network containing all types of edges. **E,** KM in the network without interactions between pseudogenes. KM, Kaplan–Meier.

This analysis identified three clusters (K = 3; Fig. 2A), which were distinguished by overall edge weight patterns: one with predominantly negative values (C1), a second with near-zero values (C2), and a third with highly positive weights (C3; Fig. 2B). Strikingly, Kaplan–Meier analysis revealed that patients in C1 exhibited significantly better OS compared with those in C2 and C3 (Fig. 2C). These findings suggest that the coordination state of pseudogenes may be associated with differential prognosis.

### *PG*_*net*_ stratification is robust and reproducible across datasets

To ensure that the clustering patterns observed in the *PG*_*net*_ were not artifacts of network construction or statistical noise, we implemented a series of rigorous validation steps. First, we considered whether applying LIONESS to highly variable edges could artificially inflate sample-to-sample variability and, in turn, drive clustering independent of biological signal. To address this, we repeated the analysis using two alternative network versions: (i) a complete network including all genes and their significant interactions, and (ii) a network excluding pseudogene–pseudogene (PS–PS) edges. Both networks were filtered to retain the top 1,508 most variable edges, matching the number used in the original *PG*_*net*_ analysis. Clustering based on the complete network closely recapitulated the structure and survival separation observed in the *PG*_*net*_ (Fig. 2D). In contrast, removing PS–PS edges resulted in weaker sample stratification, and the M3C algorithm identified only two clusters with no significant difference in survival (Fig. 2E). Principal component analysis (PCA) confirmed that networks lacking PS–PS edges exhibited reduced intersample variance (Supplementary Fig. S3), suggesting that pseudogene interactions capture a distinct layer of biological variability.

To formally evaluate whether clustering based on highly variable edges alone could lead to spurious survival associations, we generated 1,000 null models by randomly selecting 1,508 edges from the complete network. None of these models produced clusters with significant survival differences (Supplementary Fig. S4), reinforcing that pseudogene-driven stratification reflects a nonrandom and biologically relevant signal.

We next tested the reproducibility of our findings in an independent dataset of 1,284 B-ALL samples from the MP2PRT-ALL project (46). In this dataset, the *PG*_*net*_ consisted of 19,244 edges among 1,763 pseudogenes. Clustering of the top 25% most variable edges from this *PG*_*net*_ identified two patient groups with significantly different survival outcomes (Supplementary Fig. S5A). Interestingly, networks excluding PS–PS edges also yielded survival-related clusters in this dataset (Supplementary Fig. S5B and S5C), indicating that pseudogene interactions are not the sole contributors to prognostic stratification in this cohort.

### Differential coexpression analysis identifies 42 interactions associated with survival

To identify PS–PS edges that differentiate clusters based on survival, we performed a Wilcoxon rank-sum test on the scaled coexpression values across SSNs, comparing each edge between clusters. We also computed the median difference in edge weights between groups (e.g., median of C2 minus median of C1, C2 − C1).

**Figure 3.**
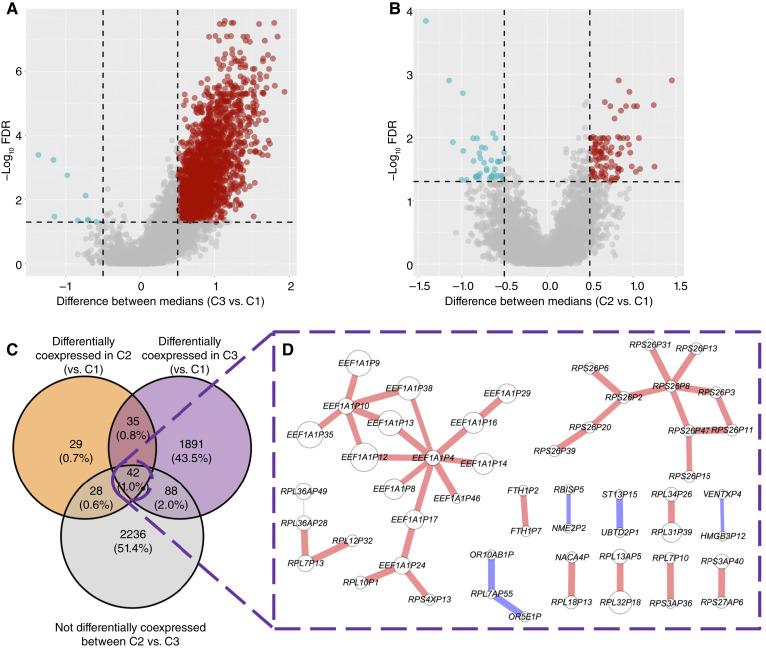
Volcano plots showing the differential coexpression analysis between clusters from the *PG*_*nets*_, (**A**) the comparison between C3 and C1. **B,** Comparison between C2 and C1. **C,** Venn diagram of differentially coexpressed edges between high (C1) and low survival (C2 and C3, orange and purple circles) clusters, vs. not differentially coexpressed edges between C2 and C3. **D,** Resulting network from the intersection in **C**. Edge color shows the differential coexpression between edges of clusters 3 V. 1. Size of nodes is proportional to their degree in the *PG*_*net*_.

We observed that the clusters associated with poorer survival exhibited overall higher coexpression scores (i.e., more positive edge weights, Fig. 3A and B; Supplementary Fig. S6). Notably, in the TARGET dataset, the C2 versus C1 comparison revealed both positively and negatively differential coexpression edges (Fig. 3A and B), indicating heterogeneity in the dysregulation patterns.

To focus on edges that robustly distinguish survival outcomes, we applied the following filtering strategy on the TARGET *PG*_*net*_:

- Edges had to show a significant difference in both the C3 versus C1 and C2 versus C1 comparisons.
- Edges had to show no significant difference between C3 and C2, as these two clusters had similar OS and differences between them are unlikely to reflect survival-associated mechanisms (Fig. 3C).

This filtering resulted in a network of 42 PS–PS interactions (Fig. 3D), which we selected for downstream analysis.

In the MP2PRT dataset, only two clusters were identified. Therefore, the differential coexpression analysis was limited to the C2 versus C1 comparison (Supplementary Fig. S6), which yielded a large number of significant edges. However, given the reduced heterogeneity in MP2PRT and the lower interpretability of its cluster-specific coexpression patterns, we focused subsequent analyses on the 42 pseudogene interactions identified in TARGET.

### Development of a pseudogene-based predictive model

So far, our analyses have shown that coexpression between pseudogenes is a widespread phenomenon in B-ALL and is significantly associated with patients’ OS. However, translating coexpression biomarkers into clinical practice remains challenging, as clinical risk stratification typically relies on variables of diverse nature, most often derived from cytogenetic or clinical assessments. A key limitation is that coexpression measurements, particularly those derived from LIONESS, are computed at the population level and are only interpretable within the context of the reference cohort used to build the network.

To obtain sample-specific coexpression values in the MP2PRT cohort that are comparable with those in TARGET, we projected each MP2PRT sample into the TARGET coexpression space (details in “Materials and Methods”). This allowed us to apply the previously 42 identified pseudogene interactions to MP2PRT in a consistent manner comparable with TARGET.

To select robust coexpression interactions for survival prediction, we combined the TARGET cohort with the projected MP2PRT samples and performed 100 rounds of LASSO-based feature selection by randomly selecting 50% of the samples.

**Figure 4.**
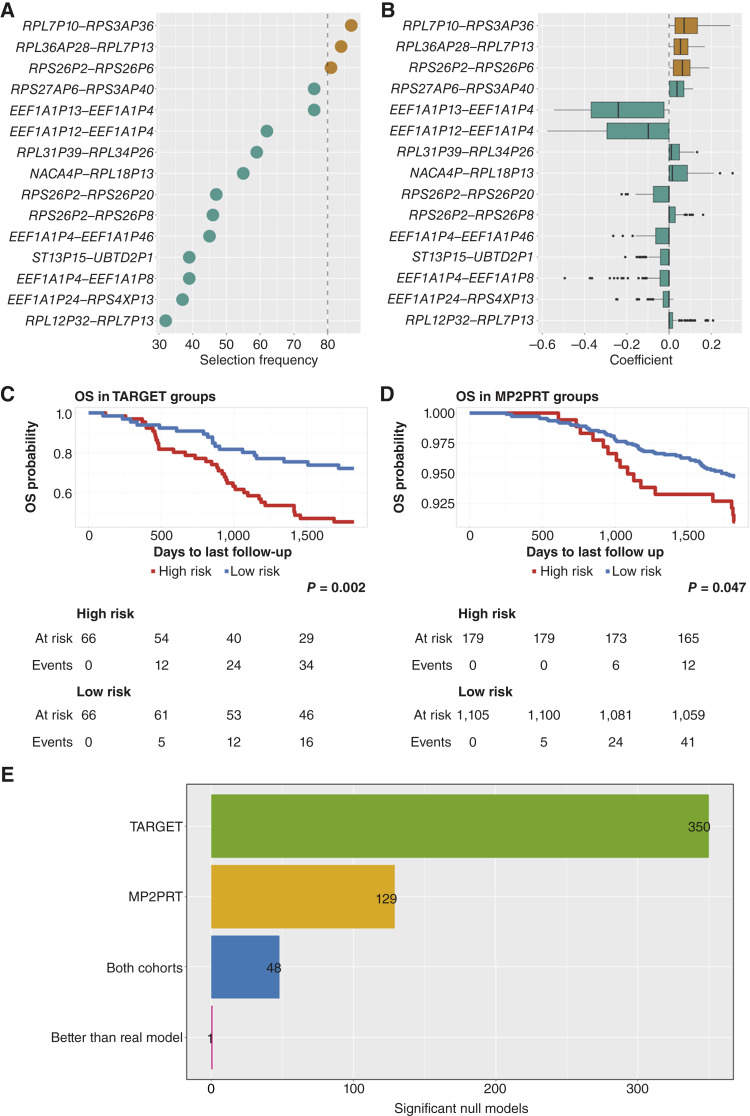
Multivariate model construction. **A,** Frequency with which each edge was selected across 100 LASSO models trained in the discovery set. The interactions selected for downstream analysis are colored in in brown. **B,** Distribution of coefficients assigned to each edge across the 100 LASSO iterations. Colored boxes indicate the three final selected features. Both panels show the top 15 interactions most frequently selected. **C,** Kaplan–Meier curve in the TARGET cohort, stratified by the multivariate model. **D,** Equivalent analysis in the MP2PRT cohort, using the same stratification threshold defined in TARGET. **E,** Amount of significant null models across different evaluation cohorts and criteria.

Next, to select the most robust and minimal set of features for the multivariate model, we ranked all candidate edges by their selection frequency across the 100 LASSO partitions. From these iterations, we identified three with high selection stability across resampling and stable coefficient values (Fig. 4A and B; Supplementary Fig. S7A and S7B). Specifically, we selected the interactions *RPL7P10*–*RPS3AP36*, *RPL36AP28*–*RPL7P13*, and *RPS26P2*–*RPS26P6* (details in “Materials and Methods”). This strategy allowed us to identify a consistent and minimal feature set under increased sample diversity, simulating a more realistic context where expression profiles may vary across patients or cohorts.

To evaluate whether the predictive signal captured by the selected pseudogene interactions generalized beyond this combined cohort (TARGET + projected MP2PRT), we trained a Cox model exclusively in TARGET (Table 1) and tested its performance in the MP2PRT data using projected LIONESS edge weights. Risk scores were computed for all samples using the trained model, and patients were classified into low- and high-risk groups based on the median risk score derived from the TARGET cohort, which served as a fixed decision threshold across cohorts. This model consistently achieved significant separation of survival curves between groups across cohorts (Fig. 4C and D), confirming that the model retains predictive value beyond the original training set (TARGET).

**Table 1.** Cox proportional hazards model trained in the TARGET dataset using three PS–PS interactions.

Edge	Coefficient (β)	HR	95% CI	*P* value
*RPL7P10*–*RPS3AP36*	0.3992	1.49	1.01–2.19	0.045
RPL7P13–RPL36AP28	0.2848	1.33	0.95–1.88	0.099
RPS26P2–RPS26P6	0.2100	1.23	0.89–1.70	0.214

Model fit metrics: Concordance index = 0.645; likelihood ratio test = 15.62 on 3 df, *P* = 0.001; Wald test = 15.05 on 3 df, *P* = 0.002; score (log-rank) test = 15.63 on 3 df, *P* = 0.001.

Next, to evaluate whether the observed cross-cohort generalization of our model could be explained by the feature selection procedure, we generated 1,000 null models by randomly selecting 42 coexpression edges from the global TARGET network (see “Materials and Methods”). Each null model was subjected to the same LASSO-based feature selection process, followed by model training exclusively in TARGET and evaluation in MP2PRT.

Among these 1,000 null models, only 4.8% achieved generalization from TARGET into MP2PRT (Fig. 4E), underscoring how rarely random feature sets yield consistent performance across cohorts, even under combined-cohort selection. Importantly, only a single null model outperformed the real model in predictive performance (Supplementary Fig. S8).

### *RPL7P10*–*RPS3AP36* exhibits consistent predictive value across multiple time points

**Figure 5.**
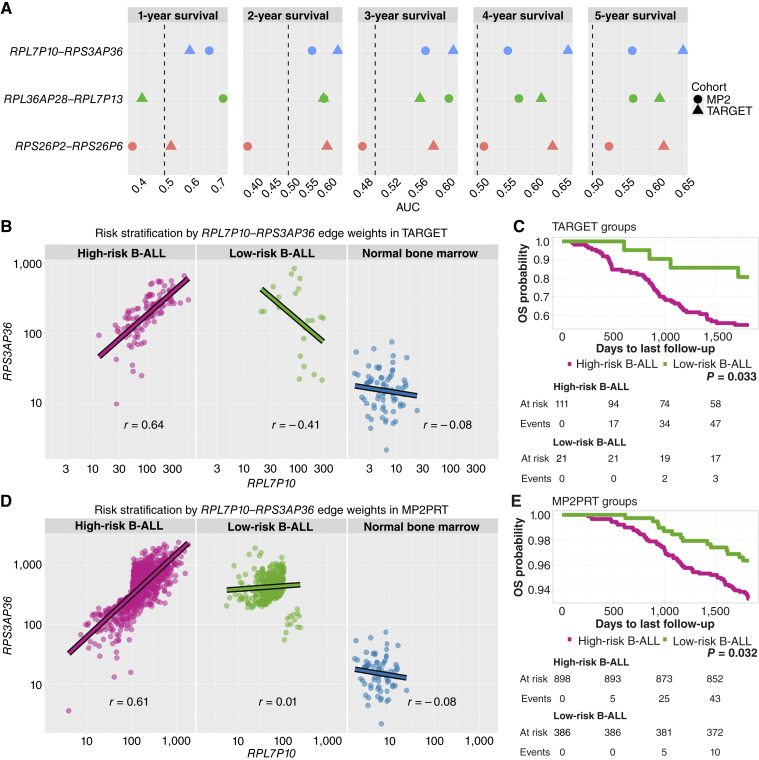
*RPL7P10*–*RPS3AP36* exhibits consistent prognostic value across cohorts. **A,** Time-dependent ROC–AUC values for the three most frequently selected pseudogene interactions in both TARGET (triangles) and MP2PRT (circles) cohorts. *RPL7P10*–*RPS3AP36* consistently maintains *AUC* > 0.5 at all time points. **B** and **C,** Risk stratification in TARGET based on the edge weight of *RPL7P10*–*RPS3AP36*. **B,** Expression relationship between *RPL7P10* and *RPS3AP36* in high-risk, low-risk, and normal bone marrow groups. Gene expression values are shown as normalized counts, as defined by the normalization pipeline for network inference described in Methods. **C,** The corresponding Kaplan–Meier curve for OS. **D** and **E,** Equivalent analysis in MP2PRT using projected data into the TARGET coexpression space. The edge weight stratifies patients into high- and low-risk groups with significant survival differences, recapitulating the patterns observed in TARGET.

To better understand the individual contribution of each component of the three interactions model, we performed a univariate evaluation of their predictive performance over time. We calculated time-dependent ROC–AUC values for 1- to 5-year OS in both the TARGET and MP2PRT (projected edge weights) cohorts. The results are summarized in Fig. 5A.

Among the three pseudogene coexpression interactions, *RPL7P10*–*RPS3AP36* consistently achieved AUC values above 0.5 across all time points and in both cohorts, indicating a stable association with patient survival. In contrast, the other two interactions showed greater variability and lower AUC values, particularly in the MP2PRT cohort.

This consistent predictive behavior of *RPL7P10*–*RPS3AP36* over multiple time horizons and across datasets supports its potential as a robust individual biomarker. Based on this observation, we selected this edge for further analysis to evaluate its prognostic value in greater detail.

To assess whether *RPL7P10* –*RPS3AP36* could independently stratify patients into prognostic groups, we classified samples based on the edge weight of this interaction. As a threshold, we used the median edge weight observed in the good prognosis group from the TARGET analysis, under the hypothesis that this value reflects a coordination state associated with favorable clinical outcomes. Using this fixed threshold, we stratified patients from TARGET and MP2PRT (using its projected edge weights) into “high-risk” (above the threshold) and “low-risk” (below the threshold) groups.

This strategy resulted in two clearly separable patient groups in both cohorts. In TARGET, the high-risk group showed a strong positive correlation between *RPL7P10* and *RPS3AP36* expression, whereas the low-risk group showed a weakly negative correlation. In MP2PRT, the high-risk group similarly exhibited strong positive correlation, whereas the low-risk group showed only weak coordination. Notably, in normal bone marrow samples, this edge showed a weak negative correlation, consistent with the pattern observed in the low-risk groups (Fig. 5B–E; Supplementary Fig. S9A and S9B). Lastly, we investigated whether the edge weights of this interaction could be associated with additional features linked to clinical outcome besides OS. For example, minimal residual disease (MRD) is considered one of the strongest independent prognostic factor in ALL (47), and integrating it with additional prognostic models could improve its predictive performance. We focused this clinical analysis in the TARGET cohort, as additional information from MP2PRT patients was not available. We evaluated the association between the *RPL7P10*–*RPS3AP36* edge weight with (i) MRD at day 29, (ii) white blood count at diagnosis (Supplementary Table S4), (iii) sex (Supplementary Table S5), (iv) age (Supplementary Table S6), and (v) ethnicity (Supplementary Table S7). In all cases, *RPL7P10*–*RPS3AP36* remained independent from the different clinical categories. Additionally, the edge weight remained a significant predictor of outcome (HR = 1.7, *P* = 0.0067) when combined with the MRD (HR = 1.02, *P* = 0.57) in a multivariate Cox model (Supplementary Table S8).

### TFBS enrichment analysis on the *RPL7P10*–*RPS3AP36* axis

Regulation of gene expression is achieved by integrating multiple structural layers of controls over a biological state. For example, TFs are central regulators of gene expression that control cellular pathways by enhancing or blocking the transcription machinery’s binding to their target DNA sequences. Regulatory elements are often seen as promising targets for novel therapies, as they can modulate the dysregulated pathways in a disease.

**Figure 6.**
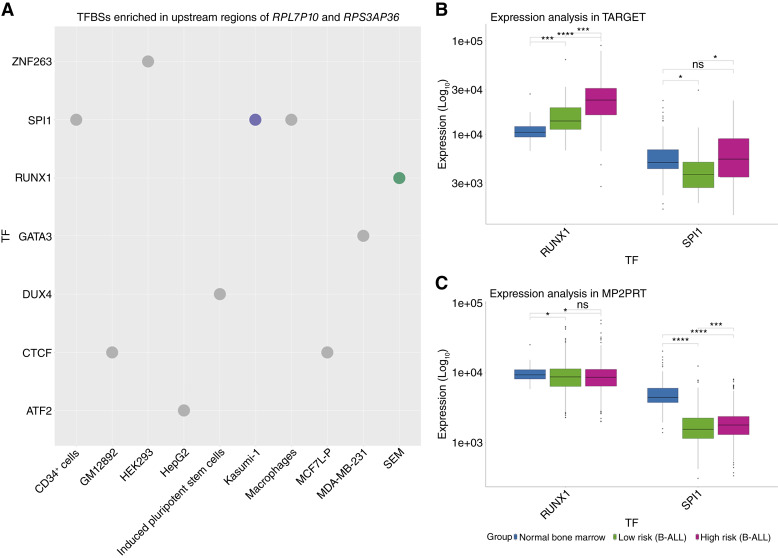
TFBS enrichment analysis for the *RPL7P10*–*RPS3AP36* interaction. **A,** Top 10 most significant enrichments on the 1,000 bp upstream and 250 bp downstream from the transcription start site. Each dot represents a chromatin immunoprecipitation sequencing experiment from the UniBind database in which binding of the indicated TF was detected in the corresponding cell line (*x*-axis). Significant enrichments (adjusted *P* value < 0.05) are colored per TF. Gray dots represent enrichments with adjusted *P* value > 0.05 (nonsignificant enrichments). **B,** Distribution of expression counts from the enriched TFs from (**A**) in the TARGET cohort. Expression values are shown as normalized TMM counts. The significance of Wilcoxon tests across groups, adjusted by multiple testing, is depicted on the brackets on top of the boxplots. Significance levels are denoted as follows: ns, not significant; *, *P* < 0.05; **, *P* < 0.01; ***, *P* < 0.001; ****, *P* < 0.0001. **C,** Distribution counts in the MP2PRT cohort as described in **B**.

Given the lack of information available for the relevant pseudogenes in our work, we explored whether they could be regulated by specific TFs. Using UniBind (43, 44), we performed a TFBS enrichment analysis (detailed in “Materials and Methods”) to identify potential regulators of the *RPL7P10*–*RPS3AP36* axis. We hypothesized that enriched TFs could influence the coexpression states of these pseudogenes and contribute to the survival outcomes observed in patients with B-ALL. This analysis revealed that the upstream DNA regions of *RPL7P10* and *RPS3AP36* are significantly enriched with binding sites for *RUNX1* and *SPI1* (Fig. 6A).

We next evaluated whether the expression of these enriched TFs was associated with the risk groups defined by the coexpression pair across datasets. In the analysis of the TARGET cohort, we found that *RUNX1* expression increased between groups (normal bone marrow → low-risk B-ALL → high-risk B-ALL). In contrast, we observed that SPI1 was underexpressed in the low-risk group in comparison with the normal bone marrow and the high-risk group (Fig. 6B). However, these differential expression patterns change drastically in the MP2PRT cohort. In the case of *RUNX1*, the distribution of expression counts greatly overlapped between groups, whereas in the SPI1 case, we found that both B-ALL groups were underexpressed in comparison with the normal bone marrow (Fig. 6C).

## Discussion

To our knowledge, this is the first study to classify patients with B-ALL solely based on coexpression interactions.

PS–PS interactions remain poorly understood. Most prior research has focused on the interactions between pseudogenes with protein-coding genes or regulatory elements. For example, Carron and colleagues (48) explored pseudogene function through coexpression with coding genes, and Chang and colleagues (49) described ceRNA networks involving pseudogenes, long non-coding RNAs, and miRNAs in lung cancer. These examples illustrate the emerging regulatory relevance of pseudogenes but leave a gap in understanding their internal coordination. Given their historic designation as “junk DNA,” we aimed to explore their potential as independent coexpression markers in leukemia.

Using data from 1,416 patients with B-ALL from the two cohorts (TARGET-ALL-P2 and MP2PRT-ALL), we constructed coexpression networks and SSNs focused on PS-PS interactions. Clustering these SSNs revealed patient subgroups with significantly different OS in both datasets, underscoring the biological relevance of pseudogene coordination.

Interestingly, *PG*_*nets*_ exhibited higher variability than networks built from other gene types, suggesting that pseudogene coexpression captures underlying molecular heterogeneity among patients. In the TARGET cohort, clustering without pseudogene edges failed to identify survival-related subgroups, whereas networks including pseudogene interactions did reveal such stratification. Although networks excluding the PS–PS interactions in the MP2PRT cohort did yield survival-associated clusters, clusters formed using pseudogene edges alone also retained predictive value, reinforcing their clinical relevance in different clinical contexts.

Differences between the results across datasets could be associated with their clinical composition. MP2PRT includes nearly 10 times more patients and is enriched for standard- or high-risk cases with genetic features associated with favorable outcome, resulting in only 6% 5-year mortality. By contrast, 40% of patients in the TARGET dataset died within 5 years. This clinical imbalance may partly explain discrepancies in the strength of the associations observed. Nonetheless, pseudogene coexpression consistently captured prognostic variation in both cohorts.

Notably, despite the clinical differences between TARGET and MP2PRT, we found consistent results across the two datasets. For example, heterogeneity in both cohorts is greatly explained by the pseudogenes coexpression (Supplementary Fig. S3), and, per our clustering analysis, this heterogeneity is linked with the patients’ OS.

Additionally, using LASSO-based feature selection across multiple random partitions, we identified a pseudogene-based, minimal and robust signature to predict a patient’s survival. Among the three interactions included in the model, the one between *RPL7P10* and *RPS3AP36* stood out for its statistical significance and stability across time points and cohorts. When stratifying patients based on this edge (using a threshold derived from TARGET), we were able to distinguish high- and low-risk groups in both datasets. High-risk patients showed strong positive correlation, whereas low-risk and normal bone marrow samples exhibited weak or negative coordination.

Although the feature selection was performed on a combined dataset of TARGET and the projected MP2PRT networks to increase variability, the final model was trained exclusively in TARGET and evaluated independently in MP2PRT. To further ensure that the model’s generalization performance was not an artifact of the selection pipeline, we generated 1,000 null models using the same training and evaluation scheme. This analysis showed that the patterns found in TARGET rarely generalize into the MP2PRT cohort (only 4.8% of models).

Overall, these results support the robustness and specificity of the multivariate model and demonstrate that the selection pipeline does not, by default, yield predictive models across cohorts. Although using data from two cohorts for feature selection could introduce information leakage into the final model, our results show that this is not sufficient to generate models with cross-cohort stability. Moreover, this indicates that the predictive power of our pseudogene-based model is mainly driven by a biological signal rather than by artifacts of the selection procedure, highlighting the utility of our strategy for selecting stable features across cohorts.

Beyond its contribution to the multivariate model, the edge *RPL7P10*–*RPS3AP36* also demonstrated independent prognostic value when used as a single-feature classifier. Using the median edge weight observed in the low-risk TARGET group (C1 in Fig. 2C) as a threshold, hypothesized to reflect a favorable coordination state, we stratified patients in both cohorts into high- and low-risk groups. This threshold yielded clearly separable groups in both datasets, with significant survival differences. High-risk groups exhibited strong positive correlation between *RPL7P10* and *RPS3AP36*, whereas low-risk groups showed weak (in MP2PRT) and mildly negative coordination (in TARGET). Interestingly, normal bone marrow samples also showed negative correlation, mirroring the low-risk profile. These findings suggest that positive coordination between this pair of pseudogenes is a leukemia-specific feature associated with adverse prognosis, whereas loss or reversal of coordination may indicate a physiologic or less aggressive state.

Importantly, our findings suggest the existence of a coordination mechanism among pseudogenes that operates independently of their expression levels and may contribute to more aggressive tumor phenotypes. In our previous work on hematologic malignancies (27), we observed that coexpression interactions predominantly occurred between pseudogenes from different chromosomes, in contrast to coding genes, which tended to interact within the same chromosome. This pattern suggests that the coordination of pseudogenes may be governed by distinct (and possibly opposing) mechanisms to those driving the loss of interchromosomal communication typically observed in cancer (bioRxiv 2022.10.27.513947v2; refs. 19, 23, 50).

A possible explanation for the coexpression (or even detected expression) of pseudogenes is a bias in RNA-seq read mapping due to high sequence similarity between pseudogenes and their parental genes. We addressed this possibility here and found no strong signs that our results were driven by this issue, supporting the biological relevance of the observed patterns.

Regarding the role of TFs in the pseudogene coexpression landscape in B-ALL, our results show that the upstream regions of *RPL7P10* and *RPS3AP36* share TFBSs for *RUNX1* and *SPI1*. Both TFs have been previously associated with leukemogenesis. Although they might be implicated in regulating the expression of these pseudogenes, our differential expression analysis showed patterns that were cohort-specific (Fig. 6). This lack of reproducibility suggests that the regulation of the *RPL7P10*–*RPS3AP36* axis might include additional elements beyond mere expression changes of the TFs, such as genomic, epigenetic, or post-transcriptional regulatory elements (51). For instance, the *ETV6::RUNX1* fusion, one of the most common fusion events in B-ALL, produces a novel protein that maintains the DNA binding motif of *RUNX1* but turns its function from a transcription activator to a repressor (52). Future research on this regard that explores multi-omics datasets is necessary to clearly elucidate the drivers and consequences of the *RPL7P10*–*RPS3AP36* coexpression interaction.

Upon further testing, these coexpression biomarkers proposed in this work could become part of novel prognostic models. Our approach to analyze the risk of new samples relative to a reference dataset provides an example of how coexpression signatures can be used to classify new patients. Future research could focus on developing coexpression biomarkers using more accessible technologies than RNA-seq, such as PCR, to facilitate their adoption in clinical settings. In addition, these new classification models could be integrated with drug repositioning approaches to recommend personalized therapies for high-risk patients, thus indirectly finding drugs that possibly target the coexpression biomarkers.

Further investigation involves exploring targeted therapies to modulate the high-risk coordination state of gene pairs. Identifying drivers of a high-risk coordination state could provide a means to alter these interactions.

Finally, integrating these perspectives into the field of network medicine could lead to novel strategies for risk stratification and therapeutic interventions. For instance, expanding traditional drug recommendation tools to incorporate coexpression and regulatory network biomarkers would enable the identification of drugs capable of rewiring a patient’s coexpression network to improve treatment response and prognosis.

### Concluding remarks

This study reveals that PS–PS coexpression captures a previously unrecognized layer of prognostic information in B-ALL. By focusing on individualized networks derived exclusively from pseudogene interactions, we identified patient subgroups associated with distinct survival outcomes across independent cohorts. Importantly, we defined a minimal pseudogene coexpression signature that stratifies patients into high- and low-risk groups and retains predictive value beyond its training cohort. Among the interactions in this signature, the one between *RPL7P10* and *RPS3AP36* emerged as a particularly robust biomarker. Together, our findings position pseudogene coexpression as a novel molecular feature with potential clinical utility for risk stratification and therapeutic development in B-ALL.

## Supplementary Material

Table S1 Distribution of gene-wise mean expression for pseudogenes and protein-coding genes. Mean expression was calculated for each gene across samples within each phenotype (BALL and normal bone marrow). The median and interquartile range (25th–75th percentile) summarize the distribution of these gene-wise mean values for each type of RNA. Values are shown in normalized RNA-seq units (see Methods).

Table S2 Correlation and expression distribution of the Top 10 co-expression interaction between pseudogenes in the TARGET network. Expression values are shown in normalized RNA-seq units (see Methods).

Table S3 Spearman correlation between expression values and sequence similarity (between pseudogenes and parental genes) by family.

Table S4 Comparison between minimal residual disease at day 29 (MRD 29) and white blood count (WBC).

Table S5 Distribution of the RPL7P10–RPS3AP36 edge weight by sex in the TARGET cohort.

Table S6 Distribution of age by RPL7P10–RPS3AP36 risk group.

Table S7 Distribution of the RPL7P10–RPS3AP36 edge weight stratified by ethnicity and risk group.

Table S8 Multivariate Cox proportional hazards models including minimal residual disease (MRD) and the RPL7P10–RPS3AP36 edge weight.

Figure S1 Correlation analysis between sequence similarity and mean expression (TPMs) of pseudogenes with their parental genes in the TARGET dataset. A)Scatter plot of p-value and correlation value of analyzed families. Families of parental genes and pseudogenes in which a significant correlation (-log10(p-value) < 2 & absolute Rho > 0.5) was found are shown in blue. B), C) and D) show scatter plots of sequence similarity (between each PS and its parental gene) and mean expression, PSs above 1 TPM are shown in red and PSs below 1 in blue. E) Proportions of members with high sequence similarity (greater than 0.5) above and below detection level (1 TPM) from the 14 families with significant correlations (A). F) Spearman’s correlation coefficient when all the members are considered (full data) versus when only members above detection level are considered; here, only families from E) in which at least 10 members were above 1 TPM were considered.

Figure S2 Correlation analysis between sequence similarity and edge weight in the aggregated network of the TARGET dataset. A to G show scatter plots by community. H) show the scatter plot of the complete network (6,032 edges).

Figure S3 Principal components analysis performed on different network subsets. Top figures show the analysis on the TARGET network, bottom figures show the analysis using the MP2PRT data.

Figure S4 Histogram of p-values from Kaplan-Meier analysis of clusters formed by randomly subsetting (1,000 times) 1,508 edges from the complete TARGET network.

Figure S5 Kaplan-Meier analysis of clusters in MP2PRT data. A) Top 25% (4,811 edges) most variable PG-PG edges B) Top 4,811 most variable edges from the network without PG-PG edges. C) Top 4,811 most variable edges from the complete network containing all classes of edges.

Figure S6 Volcano plot showing the differential co-expression analysis between clusters in the MP2PRT data formed by the analysis of the PGnets.

Figure S7 Feature stability across random partitions. A) Features were ranked by how frequently they were selected across 100 LASSO- based feature selection iterations using 50%-sampled random partitions. B) First-order differences in selection frequency between consecutive features.

Figure S8 5-year ROC-AUC values in both TARGET and MP2PRT for the 48 significant null models. Dashed lines represent the performance of the real model.

Figure S9 Coefficients of Pearson (A) and Spearman (B) correlations between samples of high and low survival risk by stratification using the single sample edge weights of RPL7P10 -RPS3AP36.

## Data Availability

Clinical and RNA-seq data corresponding to patients within the TARGET-ALL-P2, MP2PRT-ALL, and TARGET- AML projects in the GDC: https://portal.gdc.cancer.gov/. The pipeline for reproducing the results from this study is available on GitHub and can be found at https://github.com/AKNG97/PSs-SSNs-in-B-ALL.git.
