# Supplementary material for: Pseudogene Coexpression Networks Reveal a Robust Prognostic Signature for Pediatric B-ALL Survival
Source: Cancer Res Commun. 2026 Apr 16;6(4):842–56. doi: 10.1158/2767-9764.CRC-25-0706 (PMC13085861; doi:10.1158/2767-9764.CRC-25-0706)
Supplement: Table S4 — Comparison between minimal residual disease at day 29 (MRD 29) and white blood count (WBC). [file crc-25-0706_table_s4_suppst4.pdf]

Supplementary Table S4: Comparison between minimal residual disease at day 29 (MRD 29) and white blood count (WBC).

Clinical variable	Spearman ρ	Interpretation
MRD day 29	0.0359	Null association
WBC at diagnosis	-0.0141	Null association
