# Supplementary material for: Pseudogene Coexpression Networks Reveal a Robust Prognostic Signature for Pediatric B-ALL Survival
Source: Cancer Res Commun. 2026 Apr 16;6(4):842–56. doi: 10.1158/2767-9764.CRC-25-0706 (PMC13085861; doi:10.1158/2767-9764.CRC-25-0706)
Supplement: Table S5 — Distribution of the RPL7P10–RPS3AP36 edge weight by sex in the TARGET cohort. [file crc-25-0706_table_s5_suppst5.pdf]

Supplementary Table S5: Distribution of the *RPL7P10–RPS3AP36* edge weight by sex in the TARGET cohort.

**Sex**	**Median edge weight**	**IQR 25–75%**	**n**
Female	0.207	$-0.245 - 0.563$	66
Male	0.266	$-0.325 - 0.594$	66
