# Supplementary material for: Pseudogene Coexpression Networks Reveal a Robust Prognostic Signature for Pediatric B-ALL Survival
Source: Cancer Res Commun. 2026 Apr 16;6(4):842–56. doi: 10.1158/2767-9764.CRC-25-0706 (PMC13085861; doi:10.1158/2767-9764.CRC-25-0706)
Supplement: Table S6 — Distribution of age by RPL7P10–RPS3AP36 risk group. [file crc-25-0706_table_s6_suppst6.pdf]

Supplementary Table S6: Distribution of age by *RPL7P10–RPS3AP36* risk group.

**Risk group**	**Median age (years)**	**IQR 25–75%**
High risk	5.83	2.93 – 12.7
Low risk	4.53	3.35 – 10.3
