# Supplementary material for: Pseudogene Coexpression Networks Reveal a Robust Prognostic Signature for Pediatric B-ALL Survival
Source: Cancer Res Commun. 2026 Apr 16;6(4):842–56. doi: 10.1158/2767-9764.CRC-25-0706 (PMC13085861; doi:10.1158/2767-9764.CRC-25-0706)
Supplement: Table S7 — Distribution of the RPL7P10–RPS3AP36 edge weight stratified by ethnicity and risk group. [file crc-25-0706_table_s7_suppst7.pdf]

Supplementary Table S7: Distribution of the *RPL7P10–RPS3AP36* edge weight stratified by ethnicity and risk group.

**Ethnicity**	**Risk group**	**Median edge weight**	**IQR 25–75%**	**n**
Hispanic or Latino	High risk	0.589	0.265 – 0.858	23
Hispanic or Latino	Low risk	−1.09	−1.57 – −1.03	6
Not Hispanic or Latino	High risk	0.330	−0.106 – 0.540	80
Not Hispanic or Latino	Low risk	−1.25	−2.18 – −0.971	15
Unknown	High risk	0.445	0.080 – 0.963	8
