# Supplementary material for: Pseudogene Coexpression Networks Reveal a Robust Prognostic Signature for Pediatric B-ALL Survival
Source: Cancer Res Commun. 2026 Apr 16;6(4):842–56. doi: 10.1158/2767-9764.CRC-25-0706 (PMC13085861; doi:10.1158/2767-9764.CRC-25-0706)
Supplement: Table S8 — Multivariate Cox proportional hazards models including minimal residual disease (MRD) and the RPL7P10–RPS3AP36 edge weight. [file crc-25-0706_table_s8_suppst8.pdf]

Supplementary Table S8: Multivariate Cox proportional hazards models including minimal residual disease (MRD) and the *RPL7P10–RPS3AP36* edge weight.

Variable	Hazard ratio	95% CI	p-value
**Model 1: MRD only**			
MRD day 29	1.03	0.95 – 1.12	0.49
**Model 2: MRD + *RPL7P10–RPS3AP36***			
MRD day 29	1.02	0.94 – 1.11	0.57
RPL7P10–RPS3AP36	**1.70**	**1.16 – 2.51**	**0.0067**
