# Supplementary material for: Pseudogene Coexpression Networks Reveal a Robust Prognostic Signature for Pediatric B-ALL Survival
Source: Cancer Res Commun. 2026 Apr 16;6(4):842–56. doi: 10.1158/2767-9764.CRC-25-0706 (PMC13085861; doi:10.1158/2767-9764.CRC-25-0706)
Supplement: Figure S1 — Correlation analysis between sequence similarity and mean expression (TPMs) of pseudogenes with their parental genes in the TARGET dataset. A)Scatter plot of p-value and correlation value of analyzed families. Families of parental genes and pseudogenes in which a significant correlation (-log10(p-value) < 2 & absolute Rho > 0.5) was found are shown in blue. B), C) and D) show scatter plots of sequence similarity (between each PS and its parental gene) and mean expression, PSs above 1 TPM are shown in red and PSs below 1 in blue. E) Proportions of members with high sequence similarity (greater than 0.5) above and below detection level (1 TPM) from the 14 families with significant correlations (A). F) Spearman’s correlation coefficient when all the members are considered (full data) versus when only members above detection level are considered; here, only families from E) in which at least 10 members were above 1 TPM were considered. [file crc-25-0706_figure_s1_suppsf1.pdf]

Figure S1

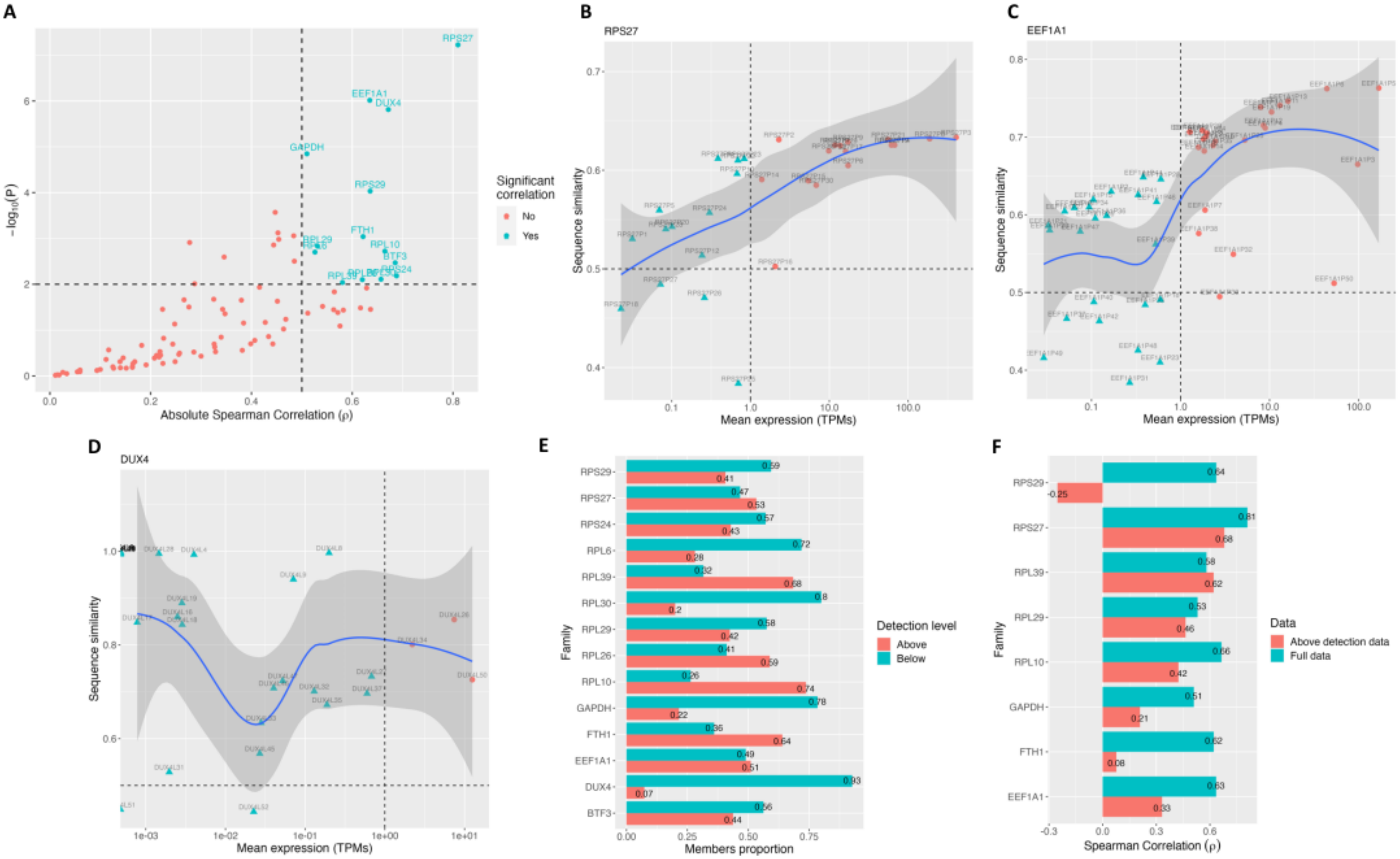

Fig. S1. Correlation analysis between sequence similarity and mean expression (TPMs) of pseudogenes with their parental genes in the TARGET dataset. **A)** Scatter plot of p-value and correlation value of analyzed families. Families of parental genes and pseudogenes in which a significant correlation (-$\log_{10}$(p-value) < 2 & absolute Rho > 0.5) was found are shown in blue. **B)**, **C)** and **D)** show scatter plots of sequence similarity (between each PS and its parental gene) and mean expression, PSs above 1 TPM are shown in red and PSs below 1 in blue. **E)** Proportions of members with high sequence similarity (greater than 0.5) above and below detection level (1 TPM) from the 14 families with significant correlations (A). **F)** Spearman's correlation coefficient when all the members are considered (full data) versus when only members above detection level are considered; here, only families from E) in which at least 10 members were above 1 TPM were considered.
