# Supplementary figures and images for: Pseudogene Coexpression Networks Reveal a Robust Prognostic Signature for Pediatric B-ALL Survival
Source: Cancer Res Commun. 2026 Apr 16;6(4):842–56. doi: 10.1158/2767-9764.CRC-25-0706 (PMC13085861; doi:10.1158/2767-9764.CRC-25-0706)

**Figure S2**

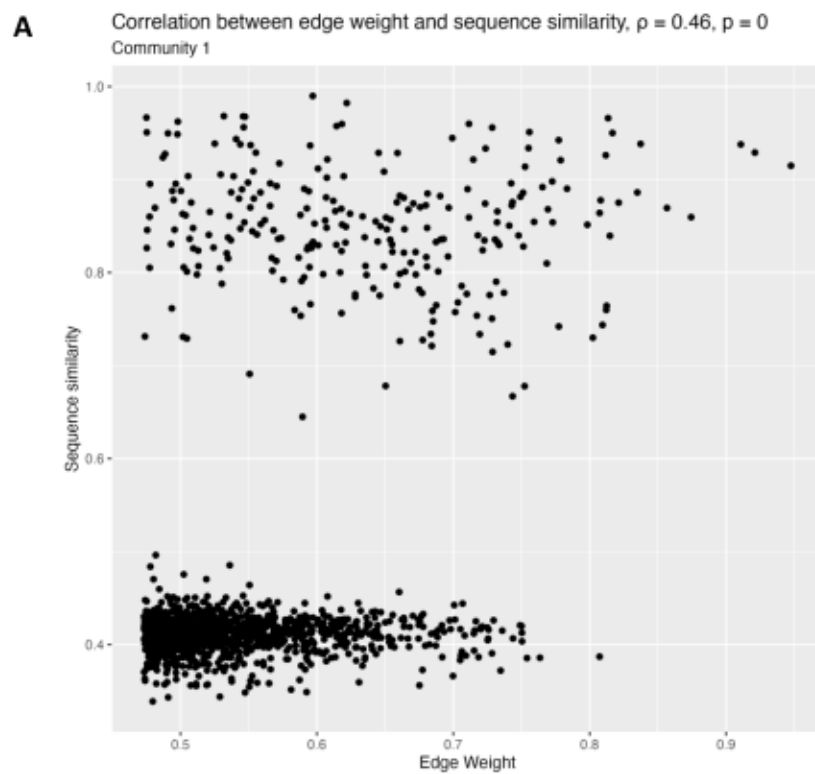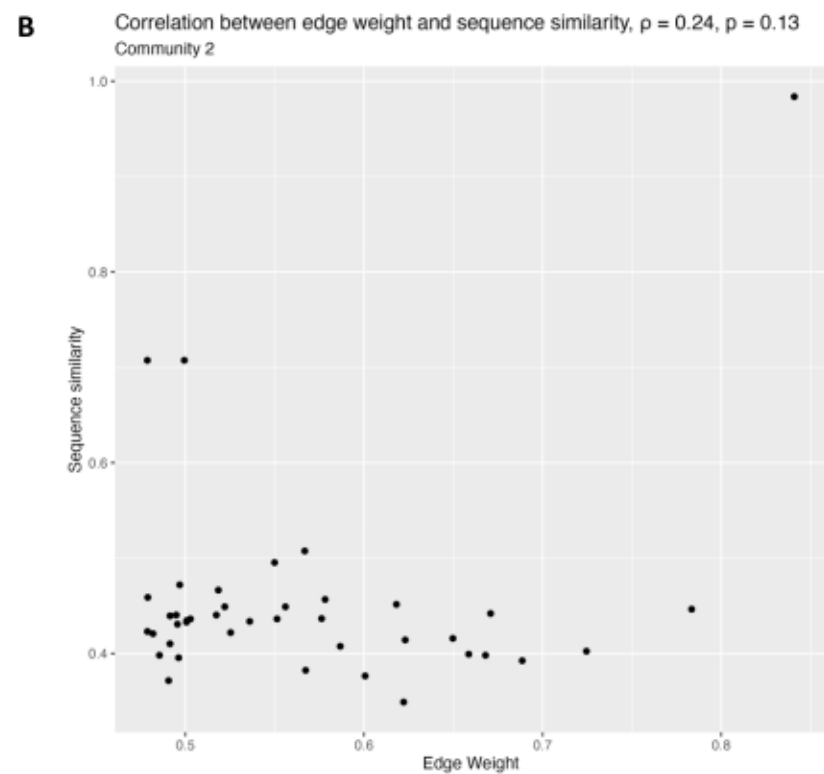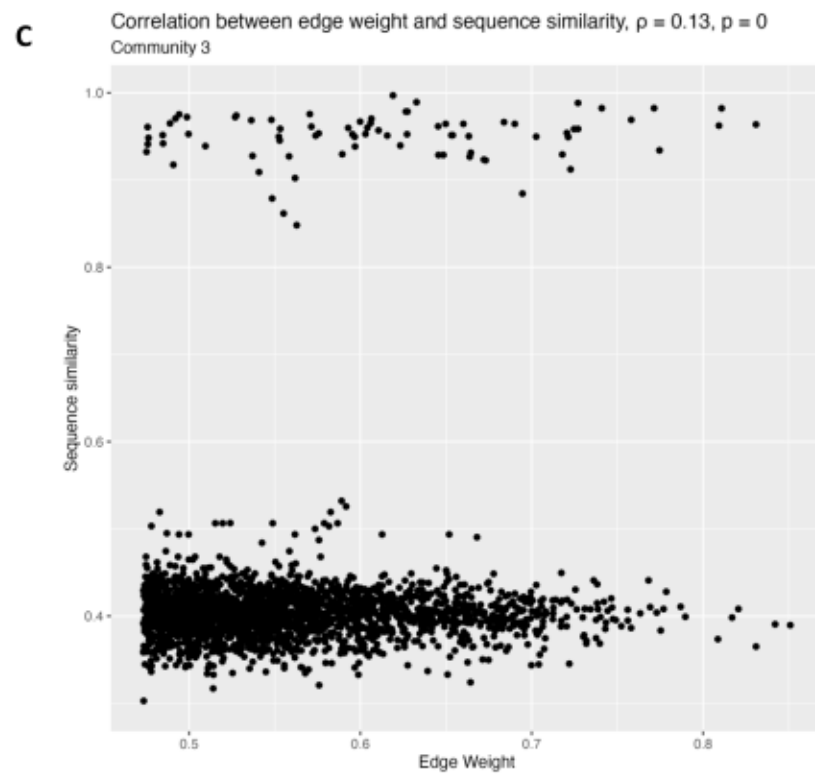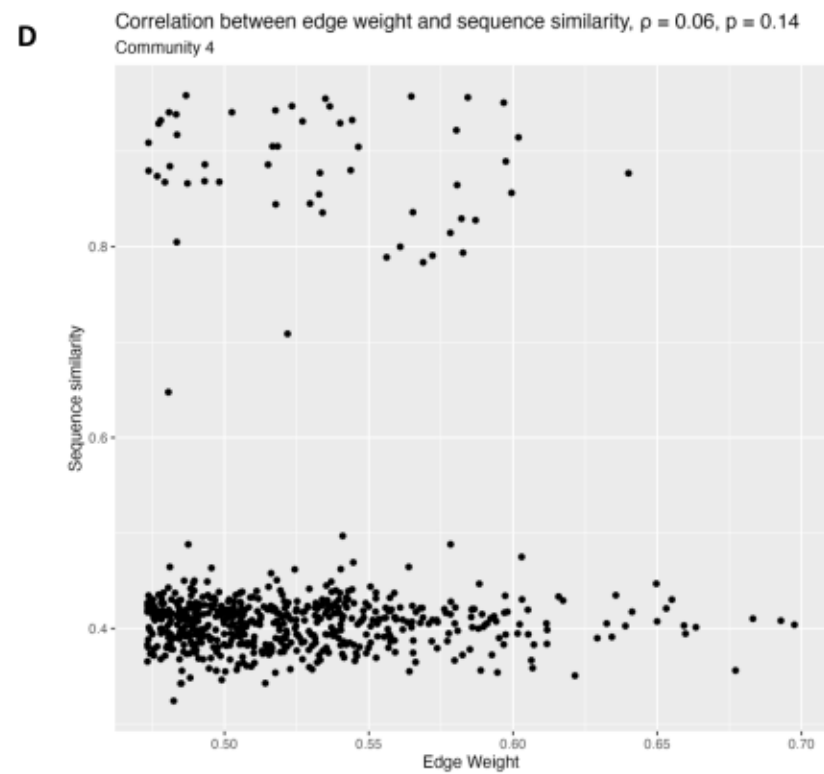

Supplement: Figure S2 — Correlation analysis between sequence similarity and edge weight in the aggregated network of the TARGET dataset. A to G show scatter plots by community. H) show the scatter plot of the complete network (6,032 edges). [file crc-25-0706_figure_s2_suppsf2.pdf]
