# Supplementary material for: Pseudogene Coexpression Networks Reveal a Robust Prognostic Signature for Pediatric B-ALL Survival
Source: Cancer Res Commun. 2026 Apr 16;6(4):842–56. doi: 10.1158/2767-9764.CRC-25-0706 (PMC13085861; doi:10.1158/2767-9764.CRC-25-0706)
Supplement: Figure S3 — Principal components analysis performed on different network subsets. Top figures show the analysis on the TARGET network, bottom figures show the analysis using the MP2PRT data. [file crc-25-0706_figure_s3_suppsf3.pdf]

Figure S3

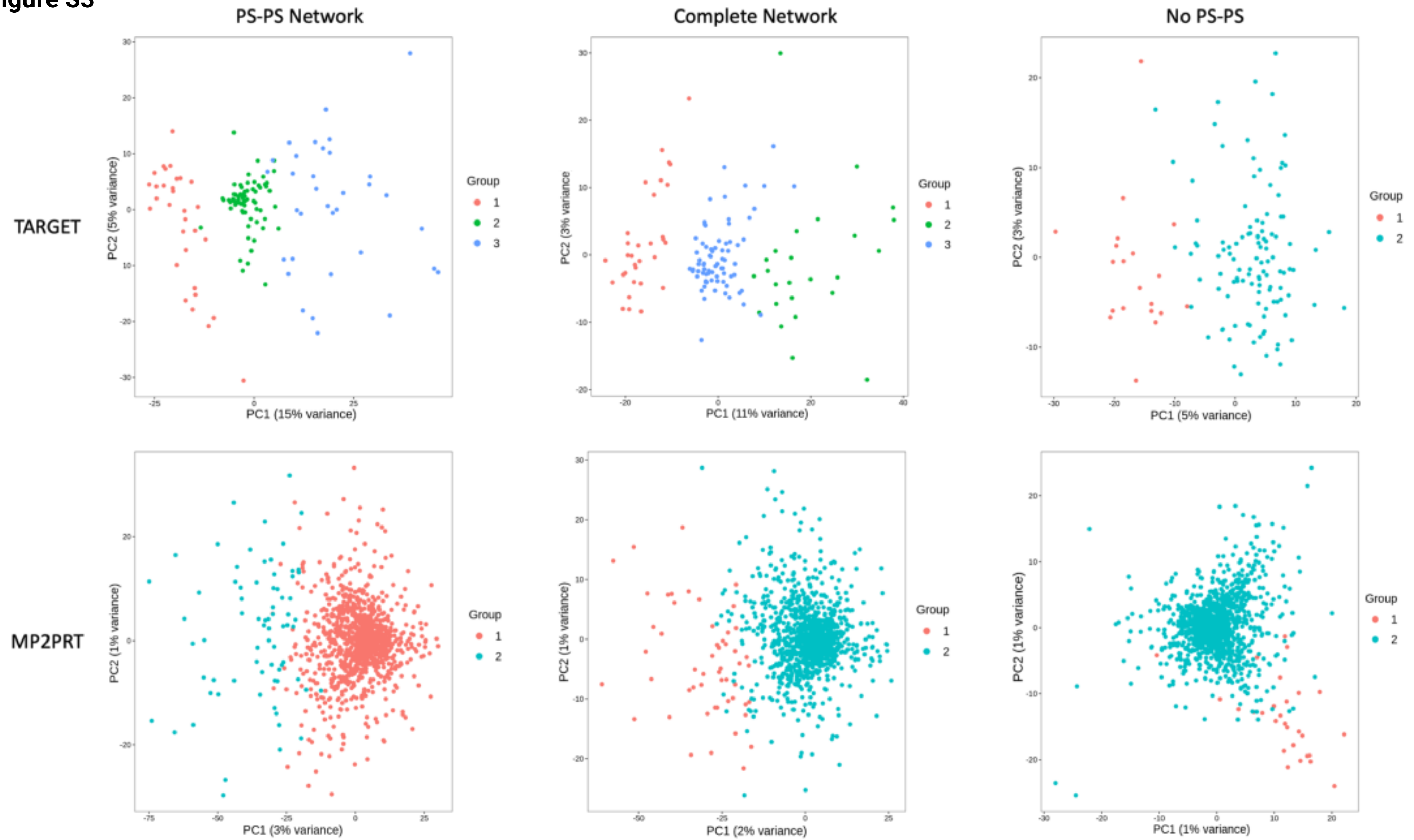

Fig. S3. Principal components analysis performed on different network subsets. Top figures show the analysis on the TARGET network, bottom figures show the analysis using the MP2PRT data.
