# Supplementary material for: Pseudogene Coexpression Networks Reveal a Robust Prognostic Signature for Pediatric B-ALL Survival
Source: Cancer Res Commun. 2026 Apr 16;6(4):842–56. doi: 10.1158/2767-9764.CRC-25-0706 (PMC13085861; doi:10.1158/2767-9764.CRC-25-0706)
Supplement: Figure S4 — Histogram of p-values from Kaplan-Meier analysis of clusters formed by randomly subsetting (1,000 times) 1,508 edges from the complete TARGET network. [file crc-25-0706_figure_s4_suppsf4.pdf]

**Figure S4**

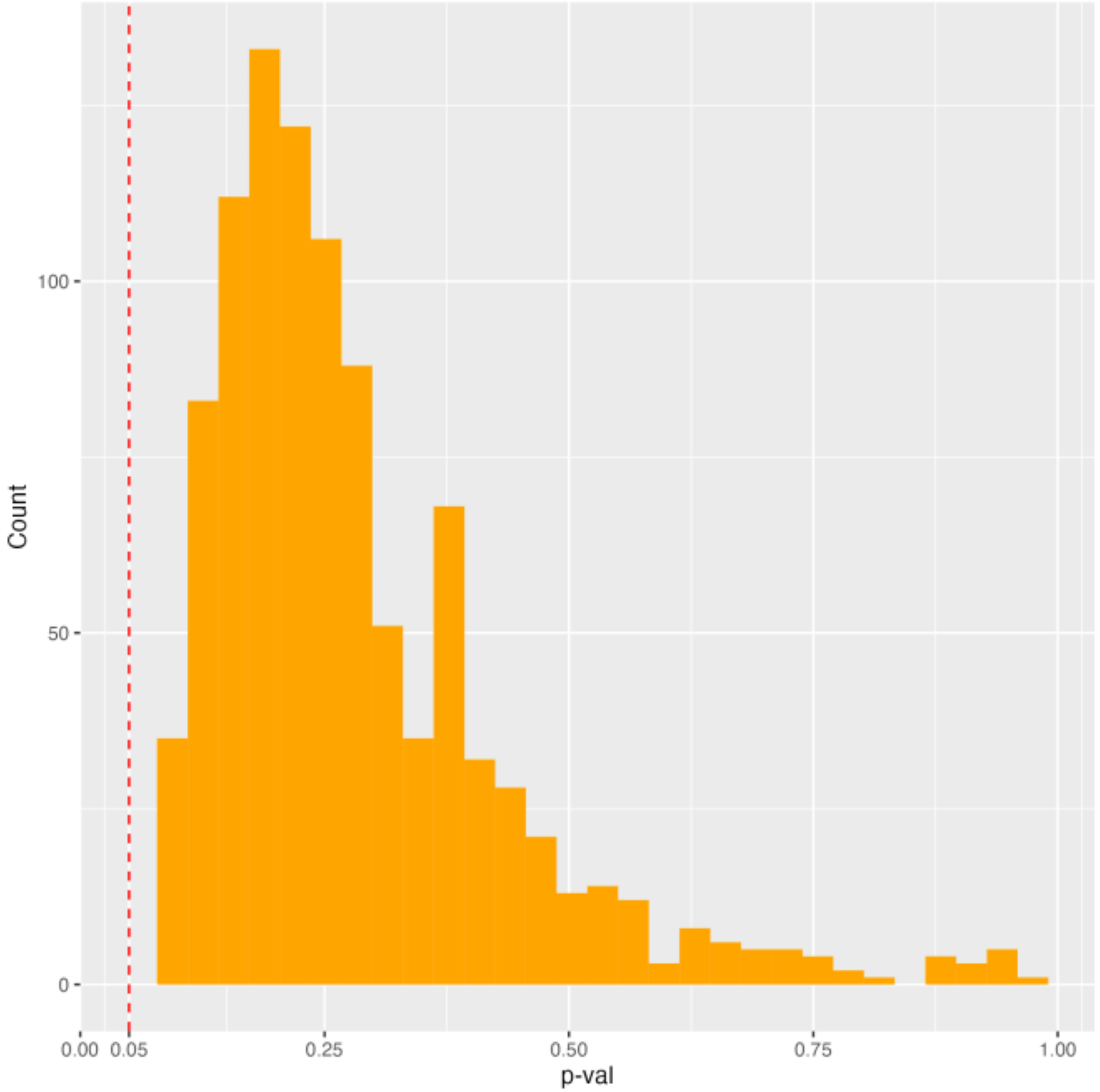


**Fig. S4.** Histogram of p-values from Kaplan-Meier analysis of clusters formed by randomly subsetting (1,000 times) 1,508 edges from the complete TARGET network.
