# Supplementary material for: Pseudogene Coexpression Networks Reveal a Robust Prognostic Signature for Pediatric B-ALL Survival
Source: Cancer Res Commun. 2026 Apr 16;6(4):842–56. doi: 10.1158/2767-9764.CRC-25-0706 (PMC13085861; doi:10.1158/2767-9764.CRC-25-0706)
Supplement: Figure S5 — Kaplan-Meier analysis of clusters in MP2PRT data. A) Top 25% (4,811 edges) most variable PG-PG edges B) Top 4,811 most variable edges from the network without PG-PG edges. C) Top 4,811 most variable edges from the complete network containing all classes of edges. [file crc-25-0706_figure_s5_suppsf5.pdf]

Figure S5

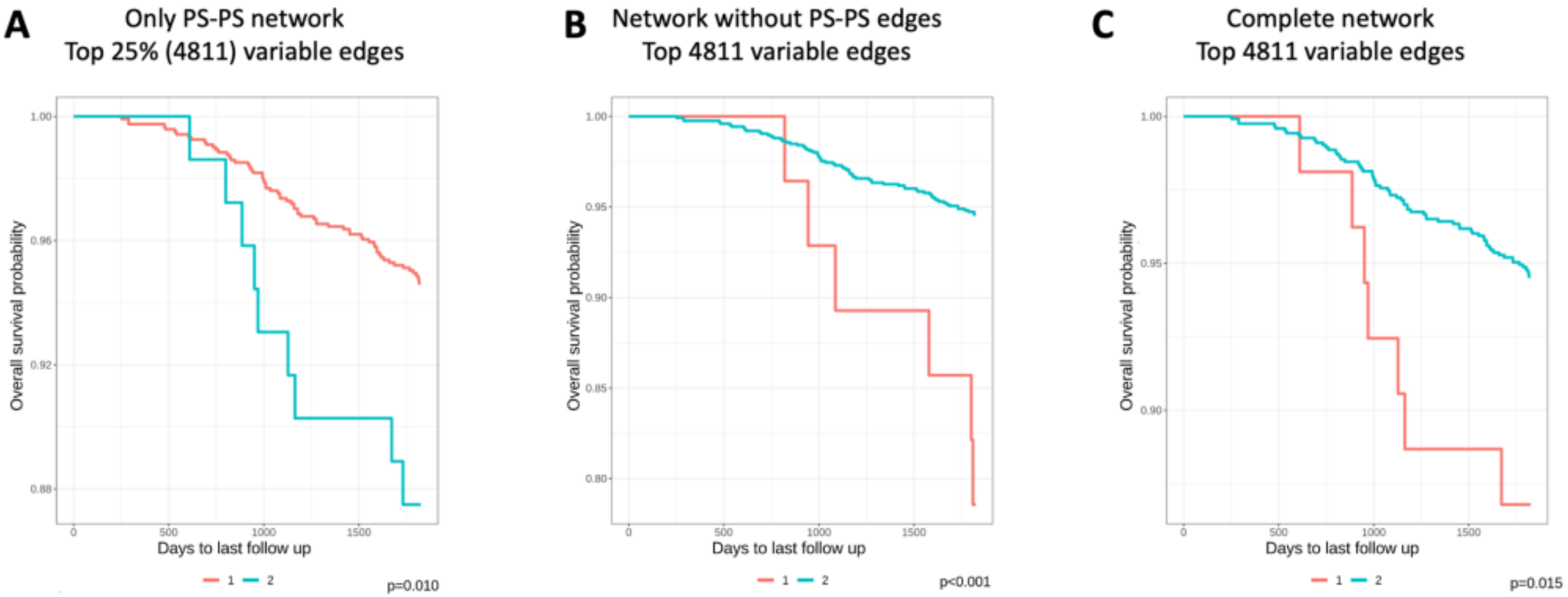

A

**1**				
At Risk	1212	1207	1187	1159
Events	0	5	25	46
**2**				
At Risk	72	72	67	65
Events	0	0	5	7

B

**1**				
At Risk	28	28	26	25
Events	0	0	2	3
**2**				
At Risk	1256	1251	1228	1199
Events	0	5	28	50

C

**1**				
At Risk	53	53	49	47
Events	0	0	4	6
**2**				
At Risk	1231	1226	1205	1177
Events	0	5	26	47

Fig. S5. Kaplan-Meier analysis of clusters in MP2PRT data. **A)** Top 25% (4,811 edges) most variable PS-PS edges **B)** Top 4,811 most variable edges from the network without PS-PS edges. **C)** Top 4,811 most variable edges from the complete network containing all classes of edges.
