# Supplementary material for: Pseudogene Coexpression Networks Reveal a Robust Prognostic Signature for Pediatric B-ALL Survival
Source: Cancer Res Commun. 2026 Apr 16;6(4):842–56. doi: 10.1158/2767-9764.CRC-25-0706 (PMC13085861; doi:10.1158/2767-9764.CRC-25-0706)
Supplement: Figure S6 — Volcano plot showing the differential co-expression analysis between clusters in the MP2PRT data formed by the analysis of the PGnets. [file crc-25-0706_figure_s6_suppsf6.pdf]

Figure S6

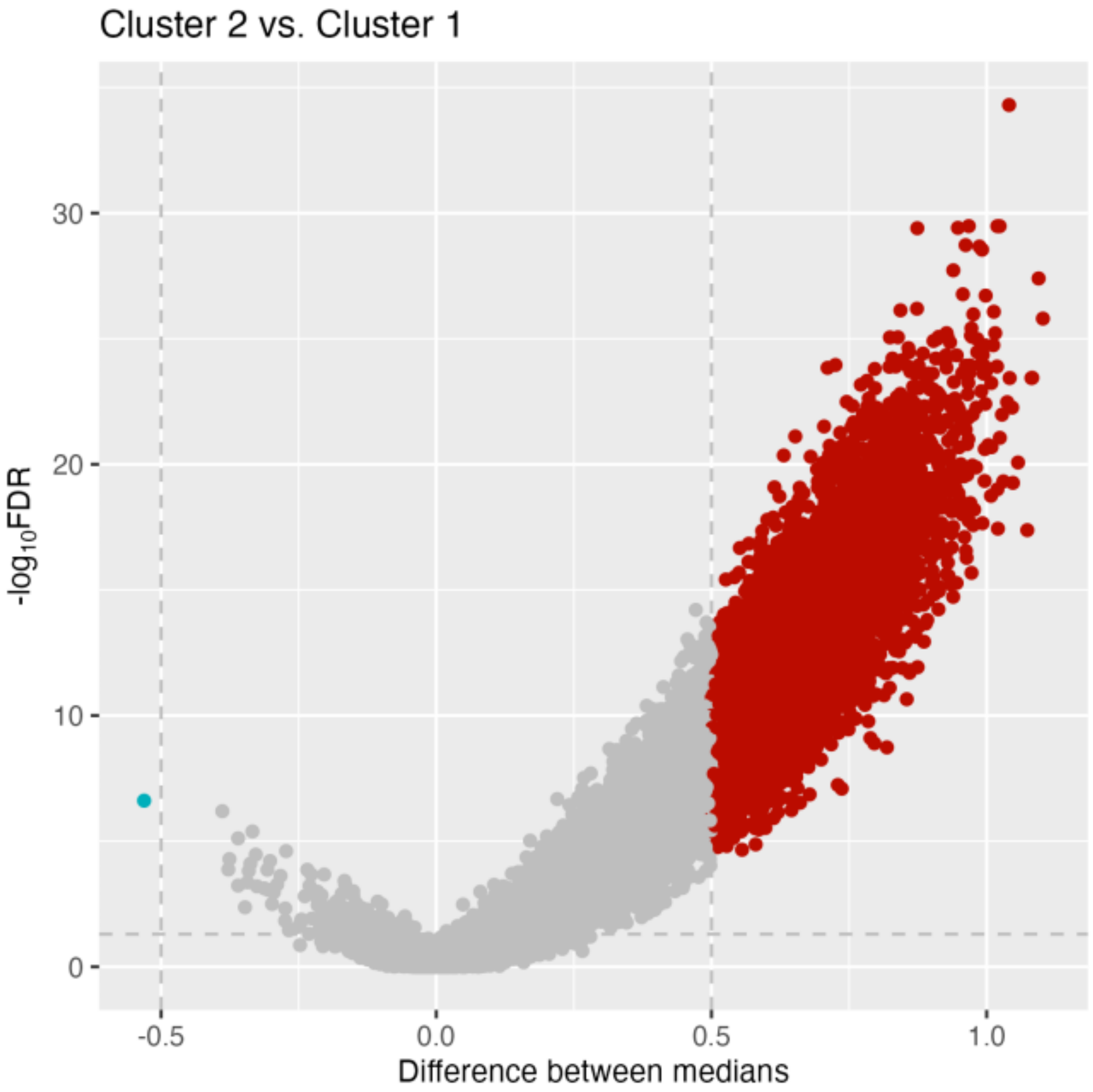

Fig. S6. Volcano plot showing the differential co-expression analysis between clusters in the MP2PRT data formed by the analysis of the PG_{nets}.
