# Supplementary material for: Pseudogene Coexpression Networks Reveal a Robust Prognostic Signature for Pediatric B-ALL Survival
Source: Cancer Res Commun. 2026 Apr 16;6(4):842–56. doi: 10.1158/2767-9764.CRC-25-0706 (PMC13085861; doi:10.1158/2767-9764.CRC-25-0706)
Supplement: Figure S7 — Feature stability across random partitions. A) Features were ranked by how frequently they were selected across 100 LASSO- based feature selection iterations using 50%-sampled random partitions. B) First-order differences in selection frequency between consecutive features. [file crc-25-0706_figure_s7_suppsf7.pdf]

igure S7

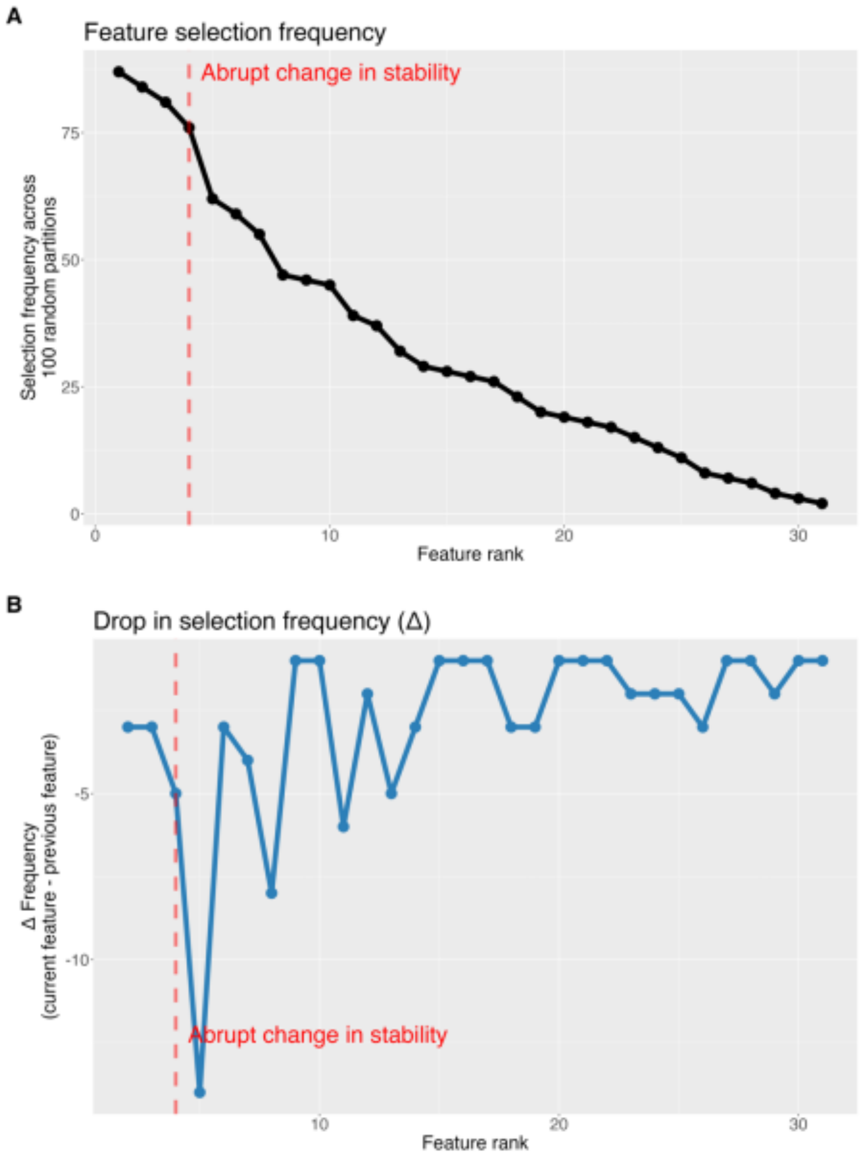

Fig. S7. Feature stability across random partitions. **(A)** Features were ranked by how frequently they were selected across 100 LASSO-based feature selection iterations using 50%-sampled random partitions. A steep drop in selection frequency after the top three features indicates a transition from highly stable to less consistently selected features. **(B)** First-order differences (Δ) in selection frequency between consecutive features show a sudden and marked drop at the same point, reinforcing the existence of an inflection point in feature stability. The dashed red line and annotation highlight this abrupt change, supporting the decision to retain only the top three most stable features for downstream modeling.
