# Supplementary material for: Pseudogene Coexpression Networks Reveal a Robust Prognostic Signature for Pediatric B-ALL Survival
Source: Cancer Res Commun. 2026 Apr 16;6(4):842–56. doi: 10.1158/2767-9764.CRC-25-0706 (PMC13085861; doi:10.1158/2767-9764.CRC-25-0706)
Supplement: Figure S8 — 5-year ROC-AUC values in both TARGET and MP2PRT for the 48 significant null models. Dashed lines represent the performance of the real model. [file crc-25-0706_figure_s8_suppsf8.pdf]

Figure S8

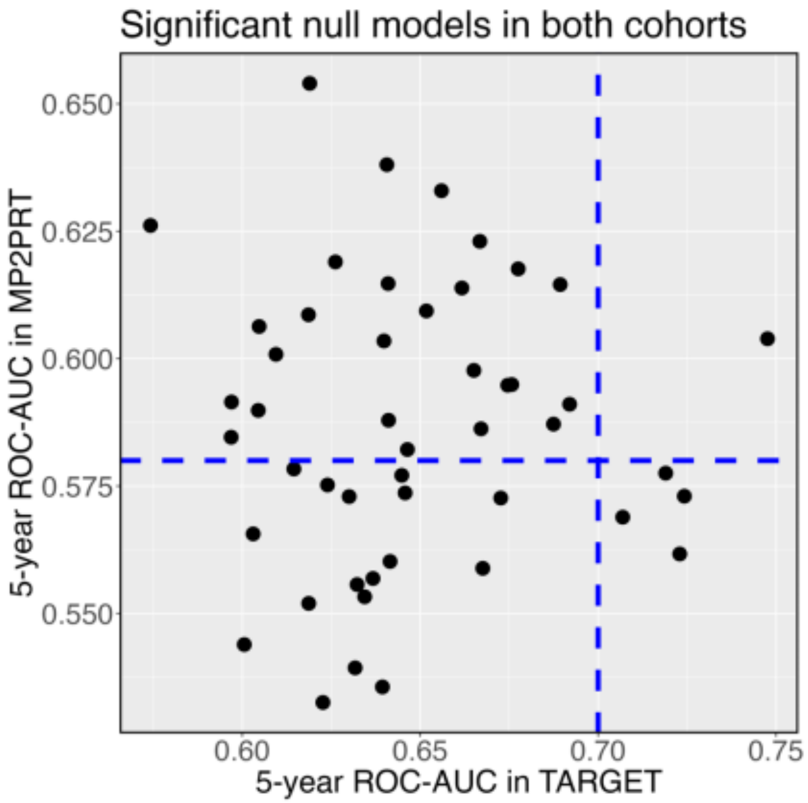

Fig. S8. 5-year ROC-AUC values in TARGET and MP2PRT for the 48 null models that consistently achieved significant risk stratification in both cohorts. Each point represents a null model. Dashed lines indicate the 5-year ROC-AUC values obtained by the real model in each cohort. Only one null model exceeds the performance of the real model in both datasets.
