# Supplementary material for: Pseudogene Coexpression Networks Reveal a Robust Prognostic Signature for Pediatric B-ALL Survival
Source: Cancer Res Commun. 2026 Apr 16;6(4):842–56. doi: 10.1158/2767-9764.CRC-25-0706 (PMC13085861; doi:10.1158/2767-9764.CRC-25-0706)
Supplement: Figure S9 — Coefficients of Pearson (A) and Spearman (B) correlations between samples of high and low survival risk by stratification using the single sample edge weights of RPL7P10 -RPS3AP36. [file crc-25-0706_figure_s9_suppsf9.pdf]

Figure S9

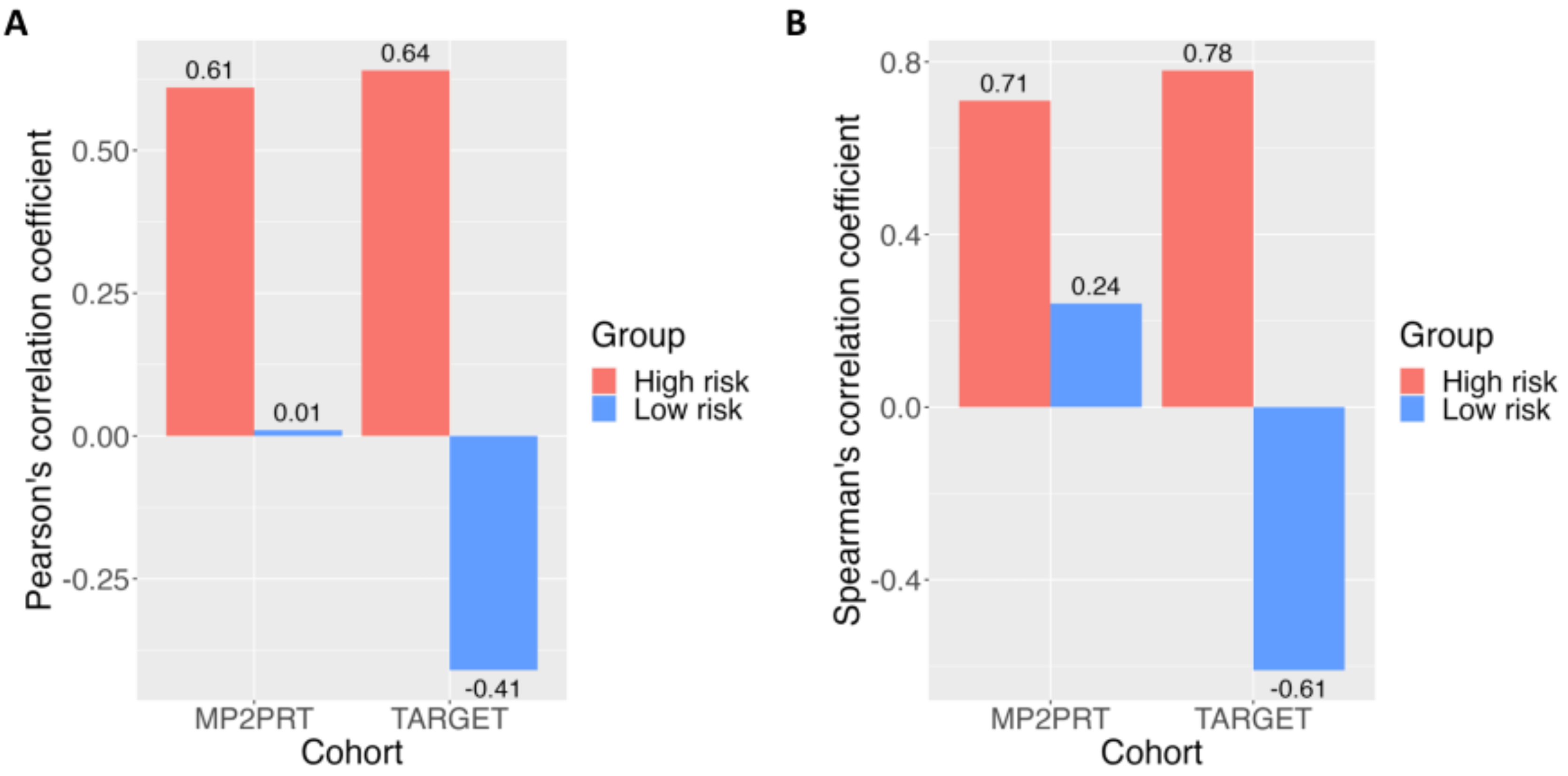

Fig. S9. Coefficients of Pearson (**A**) and Spearman (**B**) correlations between samples of high and low survival risk by stratification using the single sample edge weights of *RPL7P10-RPS3AP36*.
